# A Synthetic Facultative CAM‐Like Shuttle in C_3_ Rice Plants

**DOI:** 10.1002/advs.202500418

**Published:** 2025-02-07

**Authors:** Suting Wu, Kaining Jin, Haoshu Li, Guoxin Chen, Liying Zhang, Jinwen Yang, Shanshan Zhai, Yanni Li, Xuehui Sun, Xuean Cui, Jing Sun, Tiegang Lu, Zhiguo Zhang

**Affiliations:** ^1^ Biotechnology Research Institute Chinese Academy of Agricultural Sciences Beijing 100081 P. R. China; ^2^ Department of Plant Sciences Centre for Crop Systems Analysis Wageningen University & Research Wageningen AK 6700 The Netherlands

**Keywords:** C_3_ rice, carbon sequestration, crassulacean acid metabolism, multi‐transgene stacking systems

## Abstract

Crassulacean acid metabolism (CAM) is one of the three major forms of photosynthesis, known for its efficient carbon sequestration mechanism. CAM plants store malate at night, which undergoes decarboxylation and promotes Rubisco carboxylation during the day. Despite its potential benefits, CAM engineering is not applied to C_3_ crops. This paper introduces a designed facultative CAM bypass (CBP) in rice by incorporating codon‐optimized nocturnal carboxylation and decarboxylation modules, a malate transporter module, and a stomatal regulation module using the transgene stacking system. The CBP plants are correctly assembled by detection at the gene level, transcription level, protein level, and enzyme activity. Malate, CAM metabolism product, accumulated significantly at night in CBP plants. Metabolic analysis revealed that the malate is directed to the citric acid cycle and impacted carbon sequestration. The CBP plants showed a significant increase of ≈21% and ≈27% in photosynthetic rate and carboxylation efficiency, respectively. Additionally, CBP plants exhibited ≈20% increase in grain yield and biomass over the 2‐year field trials. Unexpectedly, the water use efficiency and drought resistance do not improve in CBP plants. This study is the first to attempt CAM engineering in C_3_ and demonstrates the potential of facultative CAM carbon sequestration in rice.

## Graphical abstract

This study utilizes gene editing and multigene stacking to design a facultative CAM‐like metabolic pathway that includes a stomatal regulation module, a carboxylation module, a decarboxylation module, and a malate transport module. A facultative CAM‐like metabolic pathway is created in C₃ rice, achieving compatibility between the C₃ cycle and the CAM cycle.

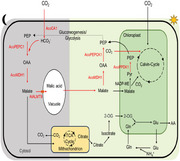

## 1 Introduction

The world population will reach 10 billion by 2050. Population increase, diminishing arable land resources, water scarcity, and the increasingly frequent occurrence of extreme weather events will seriously affect global food production.^[^ ^1^, ^2^ ^]^ Given the current pressures on population, resources, and the environment, there is a need to comprehensively increase crop yields and achieve sustainable crop production to meet the growing demands of the population. However, yields of major crops have stabilized in many regions of the world, and new technologies and solutions require exploration to increase yields.

More than 90% of dry matter is derived from photosynthesis.^[^ ^3^ ^]^ Crop yield is intricately linked to the photosynthetic process of plants, which is widely recognized as a key driver of plant growth and biomass production.^[^ ^4^, ^5^ ^]^ Improving photosynthetic efficiency is considered a promising and emerging approach to optimize crop productivity. Plants have evolved three main photosynthetic metabolic pathways, such as C_3_, C_4_, and CAM. In C_3_ photosynthesis, Rubisco catalyzes the reaction between atmospheric CO_2_ and ribulose‐1,5‐bisphosphate (RuBP), forming a 3‐carbon (3‐C) molecule and ultimately producing 3‐phosphoglycerate (PGA).^[^ ^6^ ^]^ In C_4_ and CAM photosynthesis, C_4_ and CAM plants first fix atmospheric CO_2_ into a 4‐C molecule, usually malate, catalyzed by phosphoenolpyruvate carboxylase (PEPC).^[^ ^7^ ^]^ The enzymatic reactions are configured in significantly different spatial and temporal arrangements between C_4_ and CAM. The air CO_2_ was fixed in mesophyll cells and the synthetic malate was decarboxylated in vascular bundle sheath cells in C_4._

CAM plants open stomata mainly at night for gas exchange, convert incoming CO_2_ to malate, and store malate in vacuoles at night.^[^ ^8^ ^]^ During the day, when the stomata are closed, malate is moved from the vacuoles into the cytoplasm and decarboxylated, releasing CO_2_. This is mediated by malic enzyme (ME type) or phosphoenolpyruvate carboxykinase (PEPCK type). The released CO_2_ is refixed back into the Calvin cycle by Rubisco, resulting in a decrease in cellular acidity.^[^ ^9^, ^10^ ^]^ CAM plants are thought to use water more efficiently than C_3_ or C_4_ plants by limiting diurnal gas exchange and reducing transpiration.^[^ ^11^ ^]^

The carbon metabolism mode of CO_2_ assimilation at night and the stomatal opening and closing mode of “day closed and night open” are considered key steps in the CAM pathway. *Mesembryanthemum crystallinum* is a facultative CAM plant that follows the C_3_ pathway under normal conditions.^[^ ^12^ ^]^ When faced with drought or salt stress, it undergoes the CAM pathway to resist adverse environments. *Mikania micrantha* is a C_3_ plant, but it can fix CO_2_ using the CAM pathway at night and C_3_ pathway during the day, making its net photosynthetic rate close to that of C_4_ plants.^[^ ^13^ ^]^ The example of *Mesembryanthemum crystallinum* and *Mikania micrantha* showed that the same plants exist the different CAM and C_3_ photosynthetic cycle pathway interconversion depending on the development period or environmental change. Core photosynthetic enzymes such as PEPC, CA, MDH, ME, PEPCK, etc., are present in C_3_ and CAM, but only their expression levels or patterns differ.^[^ ^14^, ^15^, ^16^ ^]^ This suggests that it is possible to create a CAM‐like metabolic pattern by regulating a small number of genes in C_3_ plants.

In recent years, the rapid development of new techniques in synthetic biology provides an opportunity to transplant the CAM pathway into C_3_ crops. First, high‐quality assembly of CAM plant genome, such as *pineapple*,*^[^* ^16^ ^]^ and *Cymbidium mannii*, and multi‐omics study of CAM photosynthesis will accelerate the domestication of CAM plants and engineering improvement of CAM in C_3_ crops.^[^ ^17^ ^]^ In addition, the integration of gene editing and synthetic biology methods can accelerate the application of CAM engineering.^[^ ^18^ ^]^ By using the CRISPR/Cas system, precise genome engineering can be achieved, along with the implementation of CRISPR/Cas‐mediated gene knockout/insertion and spatiotemporal gene expression modification. These methods can effectively enhance the performance of CAM genes in C_3_ hosts by optimizing their interactions with endogenous genes.^[^ ^19^, ^20^, ^21^ ^]^ Although the protein‐coding genes involved in this pathway have been well studied the transcriptional regulation mechanism has been elucidated to some extent. There is still a lack of practical examples of the assembly of the CAM pathway in C_3_ plants. In this work, we used the genetically modified stacking strategy to construct a facultative CAM‐like shuttle in C_3_ rice. The transgenic plants exhibited a dynamic diurnal rhythm similar to CAM plants, increased carbon sequestration, and remarkable metabolite changes. This study provides a reference for further implementation of CAM engineering in C_3_ crops.

## 2 Result

### 2.1 CAM Implementation in C_3_ Rice

**Figure 1:**
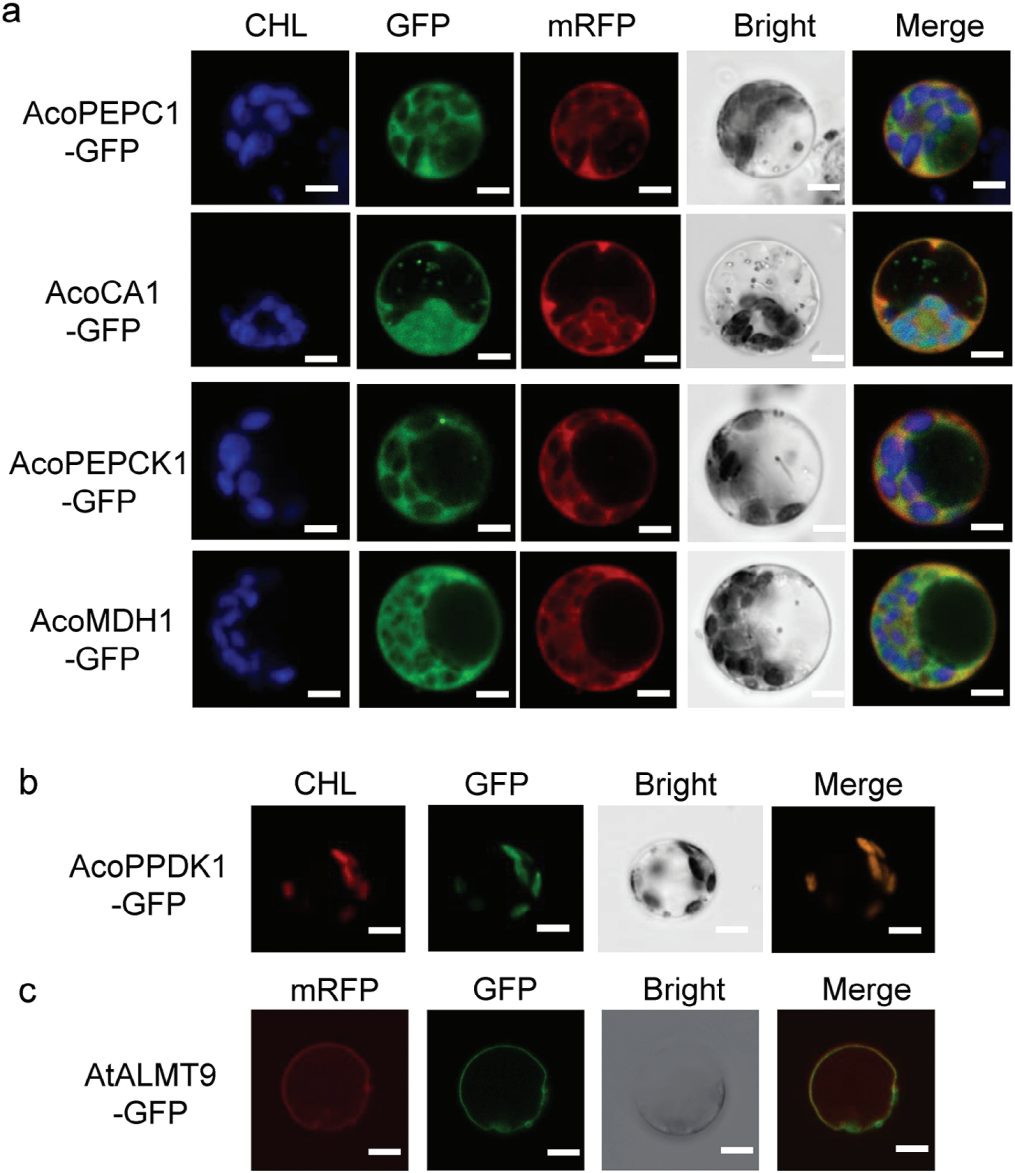
Subcellular localization analysis of the CBP bypass genes by transient expression in rice protoplasts. a) Plasmids containing the AcoCA1‐GFP, AcoMDH1‐GFP, AcoPEPC1‐GFP, AcoPEPCK1‐GFP construct were cotransfected into rice protoplasts, respectively. OsSWEET11‐mRFP fusion protein was selected as a co‐localized marker. Cells were imaged by a confocal microscopy 16h after transfection. Scale bar, 10 µm. b) Plasmid containing the AcoPPDK1‐GFP fusion was introduced into rice protoplasts. Cells are imaged by a confocal microscopy 16h after transfection. c) Plasmid containing the AtALMT9‐GFP fusion was introduced into rice protoplasts. Vacuolar Membrane Localization Plasmid containing the AtALMT9‐GFP fusion construct was introduced into rice protoplasts and co‐localization with a vacuolar membrane localization marker. GFP, GFP fluorescence; CHL, chlorophyll A autofluorescence; mRFP, the marker of cytoplasmic orvacuolar membrane; Bright, bright‐field image; Merged, superimposition of GFP, chlorophyll and/or themarker of cytoplasmic or vacuolar membrane. Scale bar, 10 µm.

We have designed a synthetic facultative CAM bypass (CBP) in rice that contains a nocturnal carboxylation module, a daytime decarboxylation module, a malate transporter module, and a stomatal regulation module. First, OsSLACl knockout mutant contributed to improving photosynthetic efficiency in rice without any adverse effects under normal conditions.^[^ ^22^ ^]^ Thus, we selected *OsSLAC1 (LOC_Os04g48530)* to create stomatal regulation module. *OsSLAC1* knockout lines using the CRISPR‐Cas9 tool were generated and two lines (*slac1‐1, slac1‐2*) showed the different deletions in the first exon of *OsSLAC1* (Figure S1a, Supporting Information). Lines *slac1‐1* and *slac1‐2* showed normal plant height and growth, similar to the wild type (Figure S1b, Supporting Information). When *slac1‐1* and *slac1‐2* were subjected to drought treatment, the *slac1‐1* and *slac1‐2* plants displayed a rolled leaf phenotype due to rapid water loss (Figure S1c, Supporting Information). The *slac1‐1* was selected as a receptor. Second, the nocturnal carboxylation module and the daytime decarboxylation module of pineapple and the aluminium‐activated malate transporter (*AtALMT9*) of *Arabidopsis* were assembled using the multi‐transgene stacking system (Table S1, Supporting Information). Notably, pineapple contains 38 genes (http://pineapple.angiosperms.org) that may be involved in CAM carbon fixation, including carbonic anhydrases (AcoCAs), phosphoenolpyruvate carboxylases (AcoPEPCs), NAD‐ and NADP‐linked malic enzymes (AcoMEs), malate dehydrogenases (AcoMDHs), phosphoenolpyruvate carboxylase kinases (AcoPEPCKs), and pyruvate phosphate dikinases (AcoPPDKs)^[^ ^16^ ^]^ (Table S2, Supporting Information). We queried the expression pattern of these genes in the pineapple database (http://pineapple.angiosperms.org) and found that the expression of *AcoCA1, AcoPEPC1, AcoPPDK1, AcoPEPCK1*, and *AcoMDH1* showed relatively high expression levels in leaves. *AcoCA1, AcoPEPC1* were induced by darkness and regulated by the circadian rhythm (Figure S2, Supporting Information). Therefore, we selected the carboxylation modules including *AcoCA1, AcoPEPC1*; the decarboxylation modules including *AcoMDH1, AcoPEPCK1, and AcoPPDK1*. Since the promoter regions of these genes may contain many cis‐regulatory elements, such as those related to the biological clock, we opted to use their native promoters to drive their expression. (Figure S3, Supporting Information). To analyze the transient expression of the six genes involved in the designed facultative CAM bypass, we cloned the coding sequences of these genes and fused them into the pAN580 vector to complete the six‐fusion protein construct: p35S::AcoCA1:GFP, p35S::AcoPPDK1:GFP, p35S::AcoMDH1:GFP, p35S::AcoPEPC1:GFP, p35S::AcoPEPCK1:GFP, p35S::AtALMT9:GFP. Transient transformations were performed in rice protoplasts, and the subcellular localization of the fusion protein was then observed using laser confocal microscopy. As shown in **Figure** **1**, the AcoPEPC1‐GFP, AcoMDH1‐GFP, and AcoPEPCK1‐GFP fusion proteins were merged with the cytoplasmic marker OsSWEET11 and expressed in the cytoplasm (Figure 1a). The AcoCA1‐GFP fusion protein was fused to the cytoplasmic marker gene *OsSWEET11* and chloroplast fluorescence, showing that it can be expressed in both the cytoplasm and chloroplast (Figure 1a). We observed that the GFP fluorescence of the AcoPPDK1‐GFP fusion protein co‐localized with the chloroplast autofluorescence (Figure 1b), and the AtALMT9‐GFP fusion protein was merged with vacuolar membrane marker and expressed in the vacuole membrane (Figure 1c).

**Figure 2:**
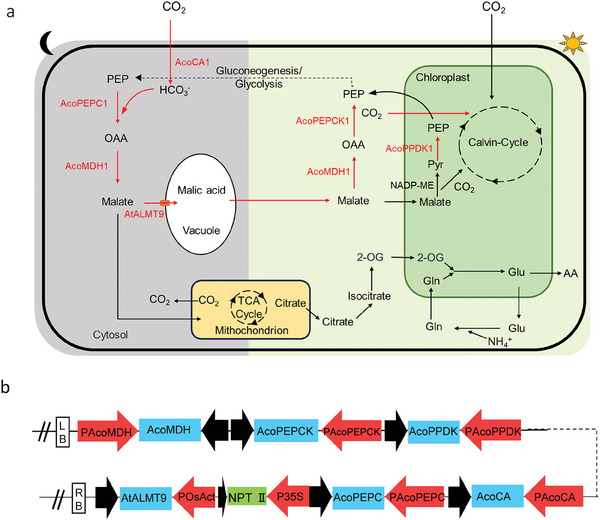
Model of the synthetic CAM bypass (CBP). a) The CAM bypass (in red) is integrated into the native rice pathway (in black).In the dark, carbon dioxide (CO_2_) is converted to bicarbonate (HCO_3_^‐^) by carbonic anhydrase (AcoCA1); this is then fixed by phosphoenolpyruvate carboxylase (AcoPEPC1), which catalyzes the formation of oxaloacetate (OAA). OAA is reduced to malate by malate dehydrogenase (AcoMDH1). Malate then enters vacuolar storage through the Vacuolar Anion Channel (AtALMT9). In the day, Pyruvate is converted to PEP by pyruvatephosphate dikinase (AcoPPDK1) in chlorophyll, or PEP generation is mediated by AcoMDH1 and phosphoenolpyruvate carboxylase (AcoPEPCK1) in the cytoplasm. CO_2_ is assimilated by Rubisco and enter Calvin cycle. The five enzymes and vacuolar anion channel introduced into rice chloroplast or cytoplasm are highlighted in red. b) Structure of the multi‐gene expression vector pCBP. LB, left border; p35S, CaMV 35S enhanced promoter; the structure of the construct containing the six target genes; the selectable marker: NPT II, neomycin phosphotransferase II;RB, right border.

**Figure 3:**
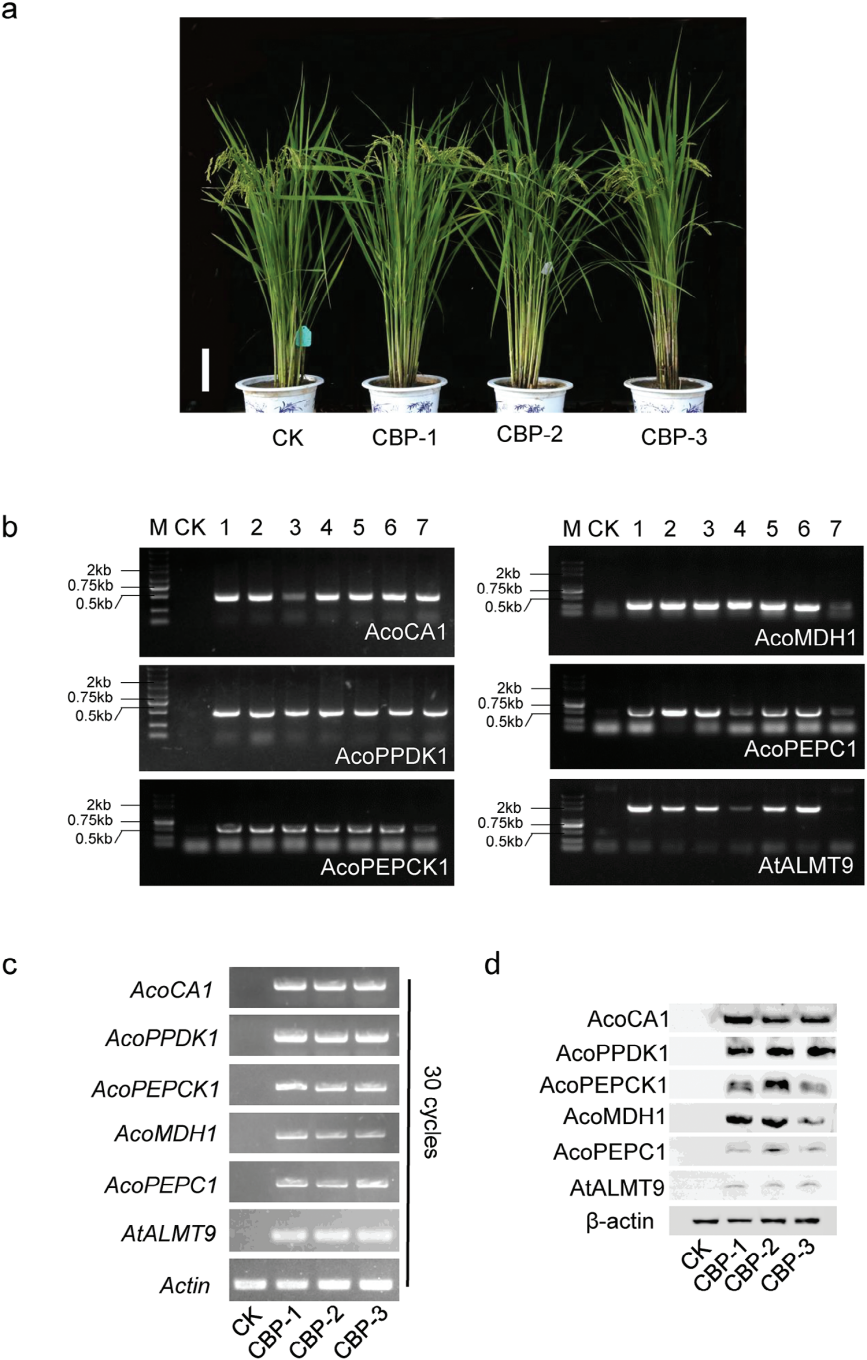
Identification of CBP lines. a) Plant architecture comparison between CK and CBP lines during the filling stage. Scale bar, 20 cm. b) The transgenes assayed by PCR in the CBP lines. c) RT‐PCR analysis utilized cDNA obtained by reverse transcription of total RNA extracted from CK leaves and three CBP plants. d) Immunoblot analysis was performed on total protein extracted from the leaves of CK and CBP plants. Rice β‐actin served as the control.

CAM plants convert CO_2_ into HCO_3_^−^ at night by the enzyme carbonic anhydrase (CA) in the cytoplasm. HCO_3_^−^ is then converted by phosphoenolpyruvate carboxylase (PEPC), using phosphoenolpyruvate (PEP) as a receptor, resulting in the production of oxaloacetate (OAA). OAA is then reduced by malate dehydrogenase (MDH) to malate, which is then transported to the vacuoles and stored as malate. During the day, when the stomata are closed, malate stored in the vacuole is mobilized into the cytoplasm and decarboxylated, releasing CO_2_, and the generation of PEP is mediated by MDH and PEPCK in the cytoplasm, while the generation of PEP in the chloroplasts is mediated by PPDK, resulting in the generation of sugars. The above carboxylation module, decarboxylation module, and malate transporter modules were assembled using the multi‐transgene stacking system (Figure S4, Supporting Information). The synthetic facultative CAM bypass (CBP) model is shown in **Figure** **2**a. The multi‐transgene stacking construct (Figure 2b) was then used to infect the SLAC1 knockout lines *slac1‐1* callus using *Agrobacterium*‐mediated transformation. Finally, 15 independent CBP homozygous lines were obtained in the T_1_ progeny and three lines (CBP1#, 2#, 3#) were selected for detailed analysis (**Figure** **3a**).

### 2.2 Analysis for CBP Lines

Expression analyses showed that the six foreign genes were well expressed in CBP lines at the DNA (Figure 3b), RNA (Figure 3c), and protein levels (Figure 3d). We also extracted cytoplasmic and chloroplast organelle proteins from the leaves of CK (empty vector transgenic plants) and CBP plants. Immunoblot analysis showed that the targeted genes localized to the predicted organelles (Figure S5, Supporting Information). All enzymes exhibited much higher activity than CK (Table S3, Supporting Information). PEPC activity was 51–61% higher in CBP lines compared to CK. PPDK activity was 9–16% higher in CBP lines than in CK. PEPCK activity was 13–32% higher in CBP lines than in CK. MDH activity was 14–30% higher in CBP lines than in CK. However, CA enzyme activity was not measured due to a lack of methods for measuring CA enzyme activity. Overall, these results showed that the CAM bypass was successfully installed in rice.

To investigate the diurnal expression of pineapple photosynthesis genes in rice, we sampled rice flag leaves every 4 h, with a cycle of 24 h, and performed qRT‐PCR analysis of gene expression patterns. The results showed that *AcoCA1* showed a nocturnal expression profile in the leaves of CBP1‐3 lines. The expression level of *AcoCA1* in CBP1‐3 lines reached its highest expression level after 2 h darkness. The high expression of *AcoCA1* in CBP1‐3 lines lasts for a long time until the morning. *AcoPEPC1* converts HCO_3_^−^ to malate and also shows a higher expression pattern at night. The AcoPEPCK1, AcoMDH1, and AcoPPDK1 exhibit a higher expression trend during the day (Figure S6, Supporting Information). This trend of these core photosynthetic CAM genes in rice is generally similar to the pattern in pineapple.^[^ ^16^ ^]^

### 2.3 Metabolite Content Determination

**Figure 4:**
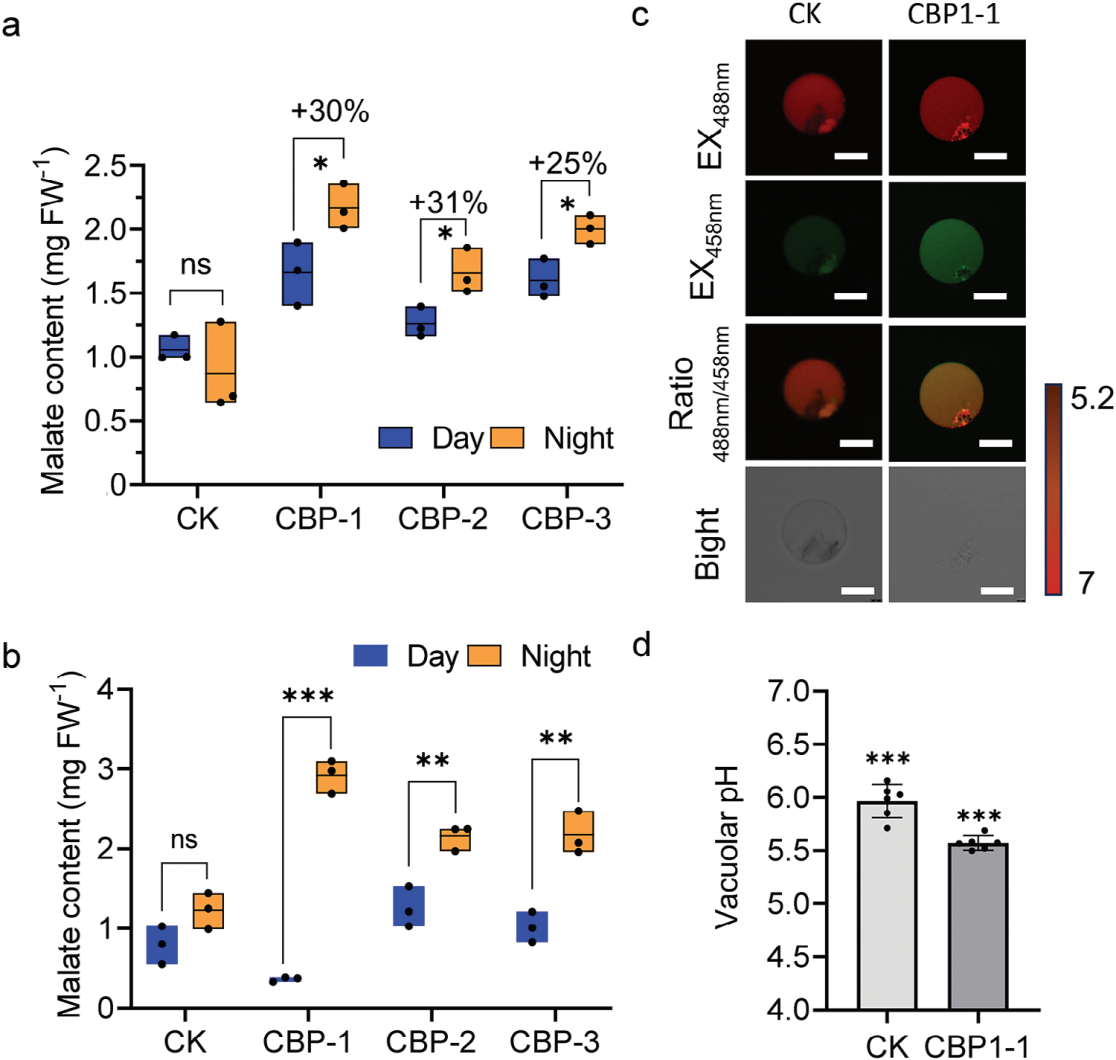
Quantification of malate content in CBP lines. a) Malate content per milligram fresh weight (FW) of titratable acid analysis of CK and CBP lines at 12:00 am (night) and  12:00 pm (day).*n* = 3. b) Determine the relative malate content per milligram of fresh weight (FW) in HPLC‐MS analysis of the CK and CBP lines at 12:00 am (night) and 12:00 pm (day). *n* = 3. c) The images show emission intensities of protoplast vacuoles in rice loaded with BCECF at 488 nm (the first column, red) and 458 nm (the second column, green). The ratio images indicate an increased or decreased vacuolar pH in rice protoplasts. The pseudo‐colored scale below indicates the fluorescence intensity. Scale bar, 10 µm. d) Quantification of the luminal pH in rice protoplasts. Bars represent from six different intact vacuoles. Asterisks represent significant differences according to Student's *t* test,^*^ *p* < 0.05, ^**^ *p* < 0.01, ^***^ *p* < 0.001. Note: Metabolite concentrations were reported as concentrations relative to the internal standard, which is the target compound peak area divided by the peak area of 2‐Aminobutyric acid: N (relative concentration)= Xi (target compound peak arc(a) *X' IS (peak area of 2‐Aminobutyrie acid per gram fresh weight.

Malate is a key biomarker of the CAM photosynthetic pathway. We use three methods to determine the content in CBP plants. First, we applied the titration method. The malate content of CBP lines at night (12:00 am) increased by 25–31% compared to day (12:00 pm). Whereas, the malate content of CK lines did not change significantly between day and night (**Figure** **4**a). Second, we used HPLC/MS to determine the malate content in the CBP lines. Compared to CK, the malate content of CBP lines was significantly higher at night (12:00 am) than during the day (12:00 pm) (Figure 4b; Figure S7, Supporting Information). Third, we used the ratiometric fluorescent pH indicator 2′,7′‐bis‐(2‐carboxyethyl)‐5‐(6)‐carboxyfluorescein (BCECF) to measure vacuolar pH^[^ ^23^ ^]^ and confirm the effects of synthetic CAM bypass on vacuolar pH. The pH values were calculated from fluorescence ratios of confocal images Figure 4c based on an in‐situ calibration curve (Figure S8, Supporting Information). As a result, the average vacuolar pH was 5.96 in CK lines, but the pH of the CBP1‐1 was 5.57 (Figure 4c,d). Hence, a higher luminal H^+^ concentration in CBP lines, indicated by the lower pH values, further supported the conclusion that the CAM bypass altered vacuolar pH. The above three experiments supported that the constructed CBP bypass accumulated malate at night, consumed malate at day and performed CAM metabolism in rice. The malate content in *Barbados aloe* (a typical CAM plant) was at least ten times higher at dawn than at dusk (Figure S9, Supporting Information). This result indicates that the constructed CAM bypass needs further optimization.

### 2.4 Determination of Photosynthetic Parameters

**Figure 5:**
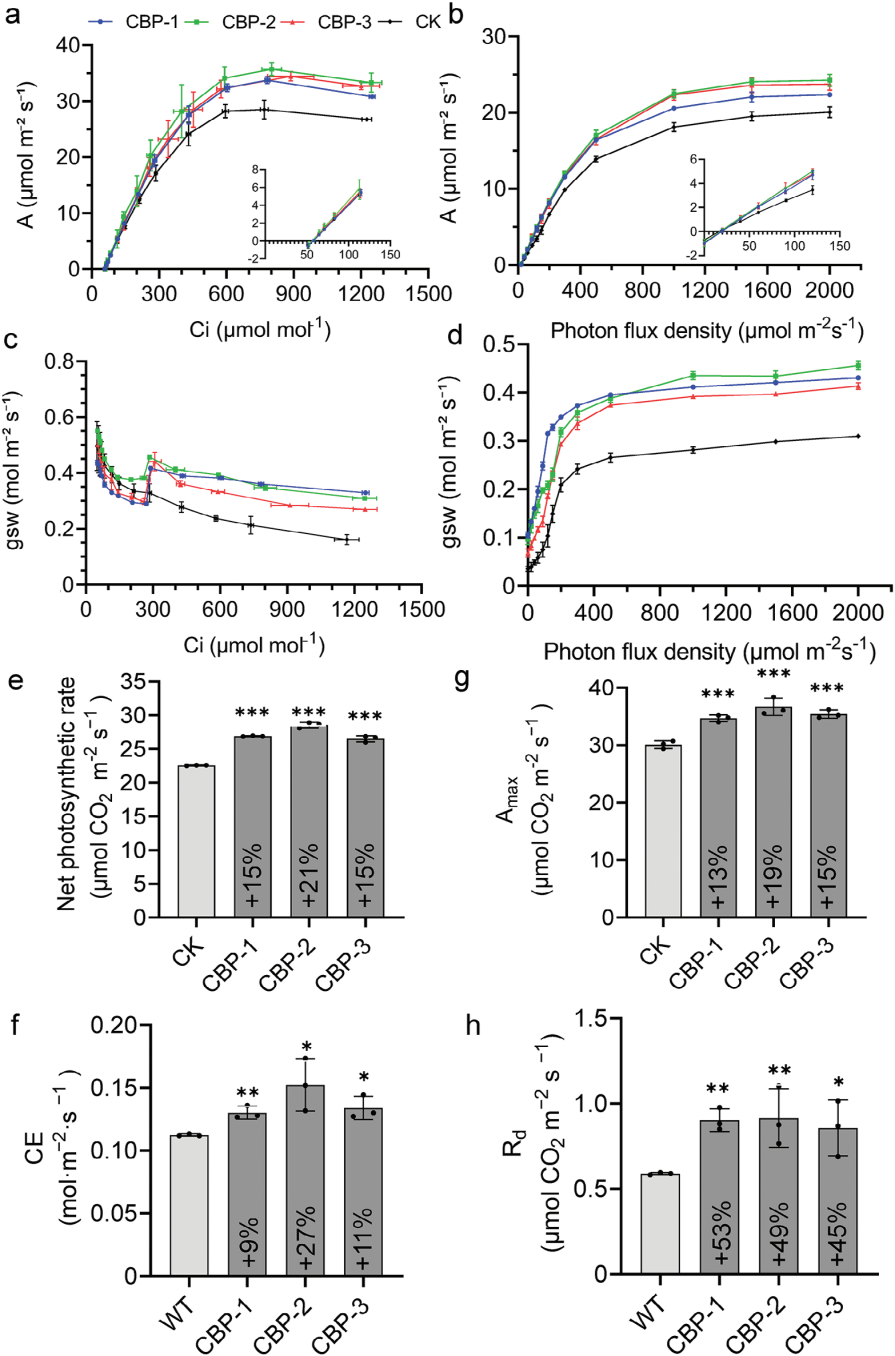
Measurement of photosynthetic parameters in the CK and CBP lines. a,b) CO_2_ response curves and Ci dependence of stomatal conductance were generated at a photon flux density (Photon flux density 1000 µmol m^−2 ^s^−1^) and a temperature of 30 °C. c,d) Light response curves and Light dependences on stomatal conductance were generated at a temperature of 30 °C under normal air conditions and a CO_2_ concentration of ≈400 ppm. e) Net photosynthetic rate was measured at photon flux density 1000 µmol m^−2^s^−1^, a temperature of 30 °C, and a CO_2_ concentration of ≈400 ppm. f) CE (carboxylation efficiency) was calculated from the slope. g,h) Amax (light‐saturated photosynthetic rate) and R_d_ (dark respiration rates) were calculated from the light‐response curves. All measurements were performed on the flag leaf of rice at the filling stage. Mean ± SD. *n* = 3; Asterisks represent significant differences according to Student's *t* test,^*^ *p* < 0.05, ^**^ *p* < 0.01, ^***^ *p* < 0.001.

To evaluate the effect of photosynthesis in CBP lines, we performed gas exchange analyses and generated CO_2_ response curves and light response curves in CK and CBP lines (**Figure** **5**a–d). The CO_2_ response curve shows that when Ci (intercellular CO_2_ concentration) is below 300 µmol mol^−1^, the CBP lines have a similar photosynthetic rate to CK. When Ci is above 300 µmol mol^−1^, the photosynthetic rate of the CBP lines increases significantly more than that of CK. When Ci increases to ≈600 µmol mol^−1^, CBP lines reach the maximum saturation point of CO_2._ The light response curves indicate that the increase in photon flux density is directly proportional to the increase in photosynthetic efficiency, both in CBP lines and the CK. When the photon flux density (PFD) increases to ≈1600 µmol m^−2^s^−1^, the CBP lines reach the maximum light saturation point. In addition, under low light conditions, the photosynthetic efficiency of CBP lines was slightly higher than that of CK lines at a photon flux density of 0 to 120 µmol m^−2^s^−1^ (Figure 5b; Figure S10, Supporting Information).

Compared to CK, CBP lines showed a significant increase in net photosynthetic rate, which was increased by 15–21% at a photon flux density of 1000 µmol m^−2^s^−1^, at a CO_2_ concentration of ≈400 ppm (Figure 5e). Carboxylation efficiency (CE) values derived from the A‐Ci curves showed a 9–27% increase in the CBP lines compared to CK (Figure 5f; Table S4, Supporting Information). This suggests that CBP metabolism may enhance the capacity to assimilate CO_2_, resulting in improved efficiency of carbon fixation compared to CK.

Light‐saturated photosynthetic rate (A_max_) was increased by 13–19% (Figure 5g; Table S4, Supporting Information). Nevertheless, dark respiration rate (R_d_) was increased by 45–53% in the CBP lines, indicating an increase in respiration rate (Figure 5h; Table S4, Supporting Information), which was supported by higher stomatal conductance in the CBP lines (Figure 5c,5h). However, stomatal conductance in the CBP lines was only slightly affected compared to CK when Ci was below 300 µmol mol^−1^ (Figure 5d). The CBP lines showed a significantly higher stomatal opening after an increase in irradiance than CK when Ci is above 300 µmol mol^−1^ (Figure 5d), leading to a higher rate of photosynthetic induction (Figure 5b).

In addition, we measured the gas exchange of CBP and CK lines after 15 min of dark treatment. The results showed that the CBP and *slac1‐1* lines had a higher stomatal conductance compared to the CK lines (Figure S11, Supporting Information).

### 2.5 Targeted Metabolite Analysis

**Figure 6:**
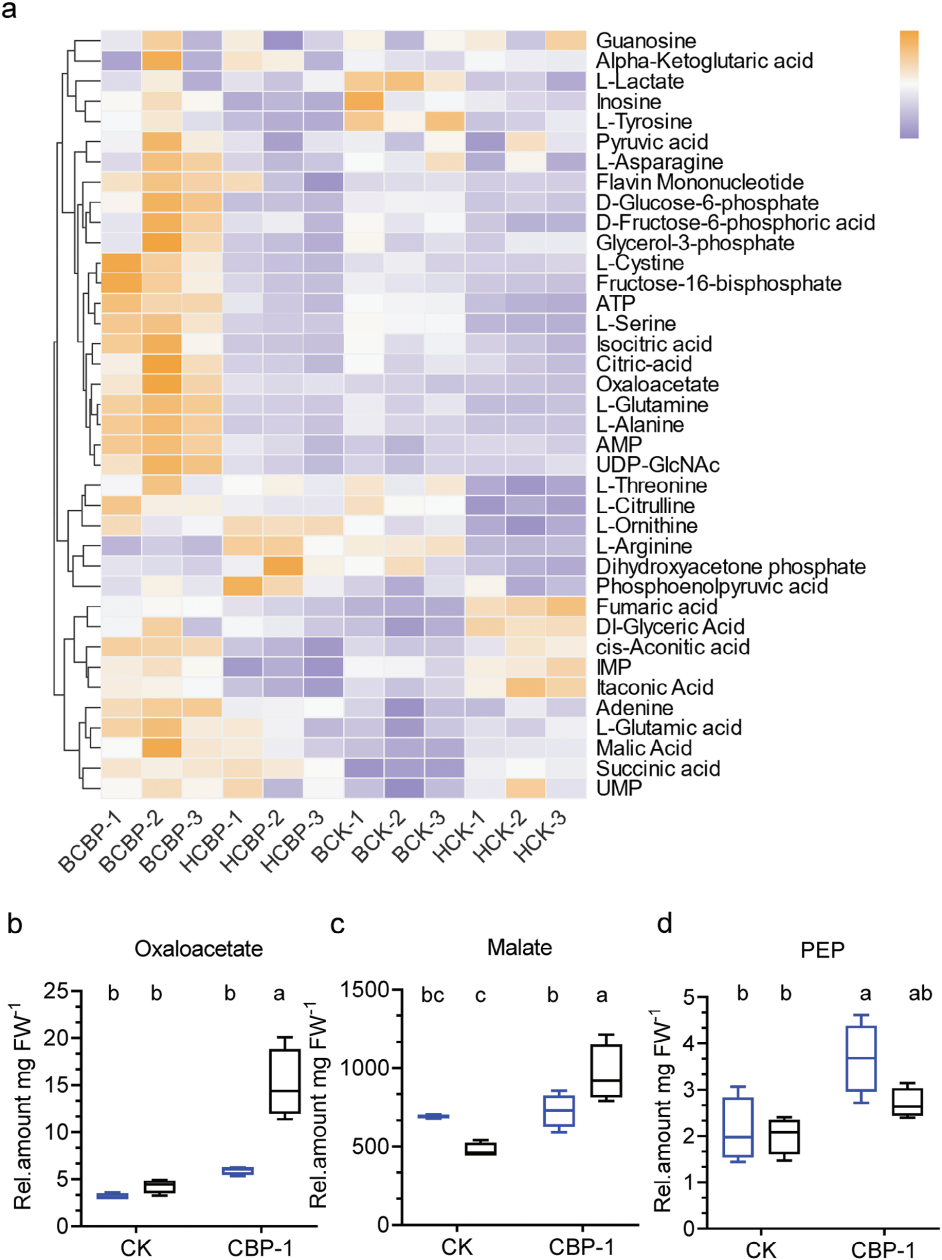
Targeted metabolomics analysis of CBP lines.a) A heat map was generated to visualize the significantly different metabolites identified among all four sample groups. BCBP represents the samples obtained at 6:00, HCBP represents the samples obtained at 18:00, BCK represents the samples obtained at 6:00, HCK represents the samples obtained at 18:00. Target metabolite content per mg FW (fresh weight) of b) oxaloacetate, c) malate and d) phosphoenolpyruvate (PEP) in CBP and CK lines. Black box represents samples obtained at 6:00 and blue box represents samples obtained at 18:00. *n* = 3. Bars (b) to (d) represent means ± SDs. Significant differences by Student's *t* test are indicated by different letters, *p* < 0.05.

Targeted metabolite analysis was performed to assess the change in metabolic flux in the CBP lines. Leaf samples from CBP1 lines and CK lines at the flowering stage were used for analysis at two‐time points: at dawn (6:00) and at dusk (18:00). The results demonstrated the presence of a total of 38 metabolites, which were grouped into four main categories: amino acids (12), organic acids (12), carbohydrates (5) and nucleotide metabolites (9). The PCA plot based on the identified target metabolites showed excellent stability and reproducibility in our current metabolite dataset (Figure S12, Supporting Information). Hierarchical clustering of the differentially abundant metabolites revealed clear differences between CBP‐1 (6:00 and 18:00) and CK (6:00 and 18:00) samples (**Figure** **6**a). Among the organic acids, a significant change was observed in malate and OAA, which is a characteristic feature of CAM plants. Malate and oxaloacetate show accumulation in CBP plants at dawn. The results showed that oxaloacetate content in CBP‐1 line increased at least two to threefold at 6:00 compared to 18:00, and malate content increased by 13–50% at 6:00 compared to 18:00, while the control group (CK) showed no significant increase (Figure 6b,c). The key metabolite PEP in the CBP bypass shows a significant increase in accumulation during the day, with levels at 18:00 being at least 1.3 to 1.9‐fold higher than at 6:00, suggesting an enhancement of the Calvin cycle (Figure 6d).

In addition, we found significant changes in the content of some metabolites in the tricarboxylic acid (TCA) cycle, such as a 1.5 to 3‐fold increase in citrate content at 6:00 compared to 18:00 in CBP‐1, a 1.5 to 2‐fold increase in isocitrate content at 6:00 compared to 18:00 in CBP‐1, and a 1 to 2‐fold increase in cis‐aconitate content at 6:00 compared to 18:00 in CBP‐1, while there was no significant increase in the control group (Figure S13a–c, Supporting Information). The accumulation of citrate, isocitrate, and cis‐aconitate in CBP‐1 indicates that the mitochondrial TCA cycle is active, and that flux into the TCA cycle is increasing.

Metabolites associated with energy metabolism, particularly in the glycolytic pathway, also show significant changes in certain key metabolites. At dawn (6:00), the levels of glucose‐6‐phosphate (G‐6‐P), fructose‐6‐phosphate (F‐6‐P), glycerol‐3‐phosphate and fructose‐1,6‐bisphosphate (F‐1, 6‐P2) showed significant increases to varying degrees in the CBP‐1 line compared to dusk (18:00) (Figure 6a; Table S5, Supporting Information). Additionally, there was a detectable increase in the content of glycolytic metabolites in CK. However, the extent of this increase was more pronounced in the CBP‐1 line than in CK.

We also found significant changes in the synthesis of amino acids. First, the levels of glutamate and its derivatives including ornithine, arginine, and glutamine changed in CBP lines and CK lines at dawn (6:00) and dusk (18:00) (Figure 6a; Table S5, Supporting Information). There were significant changes in glutamate and glutamine levels at dawn (6:00) and dusk (18:00) in CBP‐1 lines. Glutamate content increased 1.7‐fold, and glutamine content increased 2.2‐fold in CBP‐1 lines compared to CK lines at dawn (6:00) (Figure S13d,e; Table S5, Supporting Information) and there were no significant differences in glutamate and glutamine content at dusk (18:00).

Ornithine and arginine content was found to be significantly higher in the CBP‐1 lines compared to the CK lines at 18:00, with increases of twofold and 2.4‐fold, respectively (Table S5, Supporting Information). Metabolites produced from aspartate metabolism showed significant increases in CBP‐1 lines compared to CK lines at dawn (6:00), including a 1.1‐fold increase in asparagine and a 4.3‐fold increase in alanine (Figure S13f,g; Table S5, Supporting Information). Additionally, variations in the content of other amino acids, including serine and cysteine, were observed to varying degrees in the CBP‐1 lines at dawn (6:00) or dusk (18:00).

### 2.6 Biomass and Grain Yield are Significantly Increased in CBP Plants

**Figure 7:**
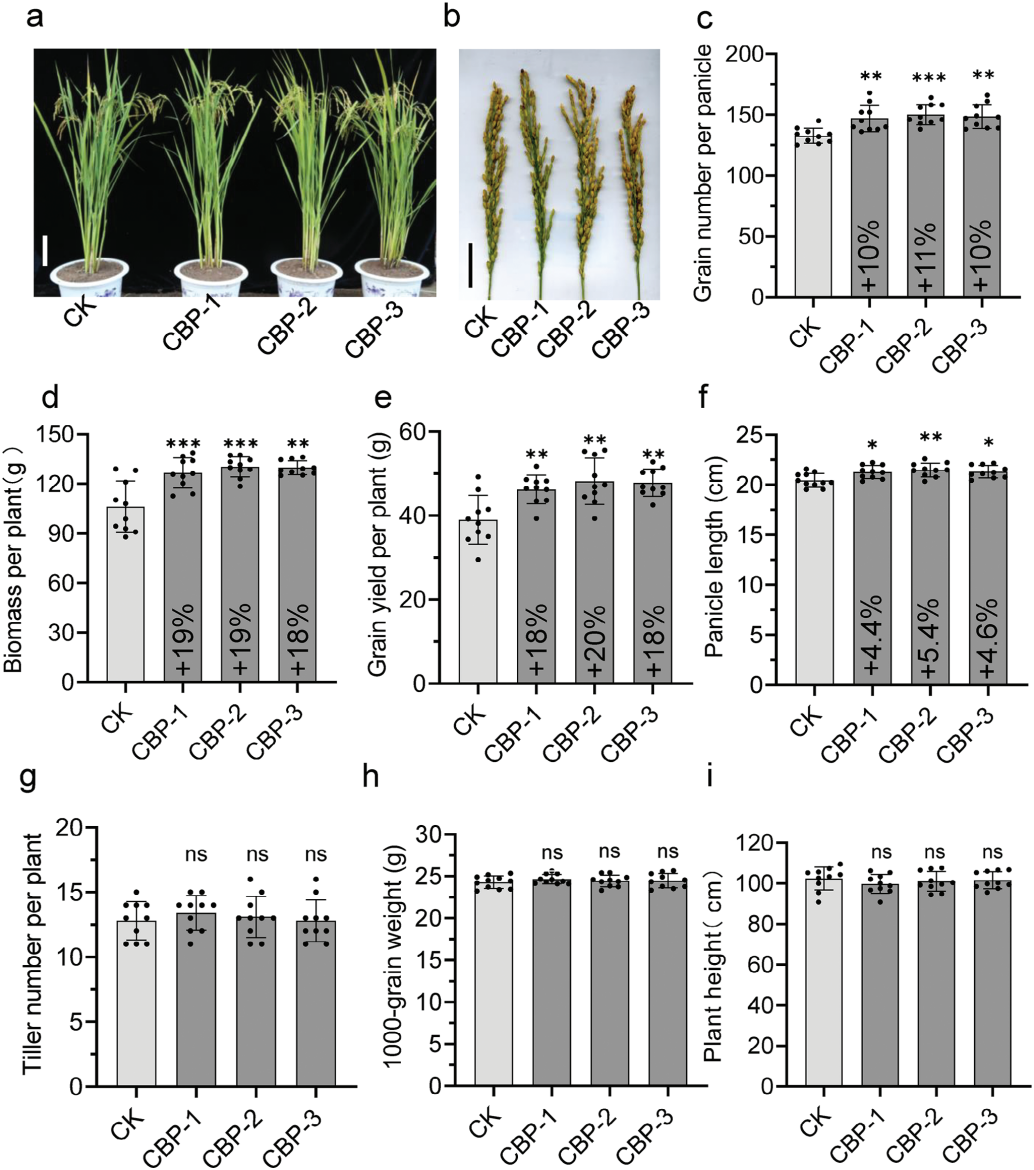
Agronomic evaluation of CK and CBP lines. a) Comparison of plant architecture between CK and CBP lines at the filling stage. Scale bar, 20 cm. b) Main panicle morphology of CK and CBP plants. Scale bar, 5 cm. c) Grain number per panicle (*n* = 10) of CK and CBP lines; d) Grain yield per panicle (*n* = 10) of CK and CBP lines; e) Biomass (*n* = 10) of CK and CBP lines; f) Plant height (*n* = 10) of CK and CBP lines; g) 1000 grain weight (*n* = 10) of CK and CBP lines; h) Tiller number (*n* = 10) of CK and CBP lines; i) Panicle length (*n* = 10) of CK and CBP lines. Ns represent no differences. Asterisks represent significant differences according to Student's *t* test, ^*^, ^**^, ^***^ significances between the CBP lines and CK at *p* < 0.05, *p* < 0.01, and *p* < 0.001 respectively.

To evaluate the effect of the CBP lines, we evaluated agronomic traits of CK and CBP lines grown in the field in 2022 (**Figure** **7**), mainly including the number of tillers, plant height, panicle length, grain yield per plant, thousand‐grain weight, number of grains per spikelet and biomass, plant architecture between CK and CBP lines during the filling stage (Figure 7a), panicle morphology of CK and CBP plants (Figure 7b). The results showed that grain number per panicle of CBP lines increased by ≈10% (Figure 7c), biomass of each plant increased by 18–19% (Figure 7d) and yield per plant increased by 18–20% (Figure 7e), panicle length of CBP lines increased by 4.4%‐5.4% (Figure 7f). However, there was no significant difference in tiller number, thousand‐grain weight, and plant height between CK and CBP lines (Figure 7g–i). Statistical data at 2023 also supported this conclusion (Table S6, Supporting Information). In our field experiment, we have incorporated CK and CBP plants to assess the impact of genetic modifications on plant growth and performance. Within each block, we have allocated nine plots for the three plant types: two independent genetically modified plant events, CBP‐1 and CBP‐2, and CK. Each plot is arranged in a 6 × 6 grid pattern, containing a total of 36 plants (Figure S14a, Supporting Information). The results showed the CBP grain yield per plot increased by 14.6–21.3% (Figure S14b, Supporting Information), and biomass per plot increased by 12.1–19.4% (Figure S14c, Supporting Information).

### 2.7 Intrinsic Water Use Efficiency in CBP Plants

Based on gas exchange data, the stomatal conductance (g_sw_) in the CBP lines was significantly higher than CK (Figure 5d), resulting in a significant decrease in intrinsic water use efficiency (iWUE) for CBP lines (Figure S15a,b, Supporting Information). To evaluate the CBP lines effect to drought, at the five‐leaf stage in the greenhouse, 20% PEG (drought simulation reagent) was used for the drought resistance test (Figure S15c, Supporting Information). After 5 days of treatment, all plants, including the control, showed a wilting phenotype. After rehydration for 7 days, an analysis of survival rates in showed no significant differences between the CBP lines and CK. These results suggested that the drought stress tolerance of CBP lines did not improve at the seedling stage. In addition, pot‐grown drought stress experiments were conducted on CK and CBP lines at the tiller stage (≈100 days). After 15 days of treatment, all plants, including the control, showed a wilting phenotype. After rehydration for 20 days, the survival rates in the CBP lines were much lower (45–55%) than in the CK lines (≈65%) (Figure S16a, Supporting Information). Dramatic reductions in both photosynthesis rates and Fv/Fm were observed in CBP lines under drought stress compared to the control group (Figure S16b,c, Supporting Information).

## 3 Discussion

CAM is one of the three major forms of carbon sequestration mechanisms in plants. In this paper, we have introduced the pineapple CAM carboxylation modules, decarboxylation modules, and malate transport module using a transgene multi‐stacking system in the rice *slac1‐1* background (the stomatal regulation module), aiming to explore the application of CAM engineering in C_3_ rice. Currently, we have constructed the facultative CAM bypass (CBP) in C_3_ rice. The successful assembly of this facultative CAM bypass in rice has been demonstrated in several ways. First, the analysis of the CBP lines showed that the six introduced genes were highly expressed at DNA, RNA, and protein levels. The expression of *AcoCA1* and *AcoPEPC1* showed a dynamic diurnal rhythm, peaking at night. The corresponding enzymatic activity from CBP flag leaves at the heading stage showed that the activity of four enzymes was maintained at a relatively high level. Second, the subcellular localization of rice protoplasts demonstrated that all six genes of CAM were correctly targeted to the designated organelles. Third, titratable acidity measurements and HPLC/MS metabolite measurements demonstrated that malate exhibited a degree of diurnal fluctuation in CBP plants, which is consistent with the expected CAM metabolic profile. The malate content was significantly higher, and the cellular pH was lower in the CBP lines compared to the CK. Fourth, gas exchange analyses showed that CBP plants exhibited higher photosynthetic efficiency and carbon sequestration capacity at high light intensities, comparable to that observed in C_4_ plants. Fifth, targeted metabolite profiling analyses have demonstrated the introduction of the CBP pathway, resulting in changes in the levels of certain metabolites in rice. Targeted metabolite analysis indicates active CO_2_ fixation, glycolysis, and TCA cycle in CBP lines. Sixth, the evaluation of agronomic traits showed that the CBP lines can significantly improve the yield and biomass of individual plants and plots. In summary, the above six aspects supported the successful assembly, expression, and execution of metabolic functions associated with the facultative CAM pathway in rice.

Introducing C_4_ or CAM pathways into C_3_ plants has the potential to significantly increase photosynthetic efficiency, ultimately leading to higher crop yields.^[^ ^24^, ^25^ ^]^ Significant efforts and achievements have been made in recent years.^[^ ^26^, ^27^, ^28^, ^29^ ^]^ Notably, South et al. developed synthetic pathways and implemented them in tobacco, resulting in an ≈40% increase in tobacco biomass.^[^ ^26^ ^]^ Peng successfully constructed a new GCGT photorespiratory bypass. The GCGT bypass redirected 75% of the carbon from glycolate metabolism into the Calvin cycle and showed a significant increase in both biomass and grain yield. This increase is mainly due to the enhanced photosynthesis caused by the increased CO_2_ concentration in the chloroplasts, similar to that in C_4_ plants. However, GCGT also showed an unstable seed‐setting rate due to poor source flow.^[^ ^27^ ^]^ Ermakova used a single construct to introduce five C_4_ pathway enzymes from maize into rice, which resulted in enhanced levels of the C_4_ proteins in rice. However, due to the lack of anatomical structure of C_4_, the C_4_ rice created did not significantly improve photosynthetic efficiency.^[^ ^28^ ^]^ Weber engineered a functional BHAC in plant peroxisomes to create a photorespiratory bypass that is independent of 3‐phosphoglycerate regeneration or the decarboxylation of photorespiratory precursors. However, the levels of the 3‐phosphoglycerate were reduced in BHAC plants, and this impairment in photorespiratory 3‐phosphoglycerate regeneration led to reduced photosynthesis.^[^ ^29^ ^]^ Compared to the above studies, our created facultative CAM‐like plants showed higher biomass and yield, owing to that malate was directed to the citric acid cycle and impacted carbon sequestration. In addition, malate produced by facultative CAM‐C_3_ plants undergoes decarboxylation to produce pyruvate and CO_2._ An increased CO_2_ concentration around Rubisco significantly increases the efficiency of the Calvin cycle.

We searched the database and found no current reports on CAM engineering practices in C_3_. This is our first successful assembly of a synthetic CAM bypass in C_3_ rice. However, the CBP plants we created were unlike typical CAM plants and more similar to *Mikania micrantha*. *Mikania micrantha* belongs to CAM‐C_3_ plant and can use different photosynthetic pathways to fix carbon dioxide during the day and night, making its net photosynthetic rate similar to C_4_ plants.^[^ ^13^ ^]^ The sufficient carbohydrate content leads to its rapid growth characteristics. CBP plants also integrate different photosynthetic pathways to fix carbon dioxide during the day and night. In fact, the overall growth of traditional CAM plants, such as pineapple, is not fast. They only have strong environmental adaptability under extreme adverse conditions, and therefore cannot lead to higher biomass and yield. CAM plants were actually a trade‐off between plant growth and stress. At present, due to the constant opening of stomata, our facultative CAM‐C_3_ design is not appropriate for drought‐resistant crops such as wheat. In the future, inducible or specific promoters will be used to regulate stomata movement, thereby creating drought‐resistant facultative CAM‐C_3_ plants.

Why wasn't the CAM bypass built into the predecessors of the C_3_? We speculate that the main reasons are as follows: CAM plants are not model plants and have a wide range of species, including xerophytic, aquatic, and facultative CAM. The large genome and complex CAM mechanisms limit CAM engineering in C_3_ plants. In recent years, with the development of sequencing technology and the reduction of sequencing costs, the multi‐omics data from CAM plants have shown an explosive trend. Significant progress has been made in the study of CAM photosynthetic core homologous genes, transcriptional regulatory elements, regulatory networks, and evolutionary trajectory analysis within CAM metabolism. This progress has promoted a deeper understanding of the CAM pathway within the academic community.^[^ ^30^ ^]^ For example, spatial and metabolic transcriptome sequencing of *Portulaca oleracea* revealed that the difference in CAM/C_4_ was not as significant as thought, and suggested that CAM/C_4_ pathways may switch between each other under different stress conditions.^[^ ^31^ ^]^ The multi‐omic dynamics of *Platycerium bifurcatum* (elkhorn fern) demonstrate the coexistence of the C_3_ and CAM pathways in different tissues, with C_3_ and CAM pathways occurring in different locations.^[^ ^32^ ^]^ This suggests that CAM localization of C_3_ can be achieved with only a few element manipulations, providing hope for the creation of CAM bypasses in C_3_ plants. Second, in recent years, the main focus has been on the functional characterization of the CAM pathway‐related genes and their relationship with the physiological and morphology traits of CAM plants, with emphasis on the regulation of stomatal movement, metabolism, and water use efficiency. However, many other issues related to CAM still require further investigation, such as the current focus of a large number of studies, which often focus on nocturnal carboxylation, while there is a lack of attention to the driving mechanism behind the mobilization of malate from vacuoles during the day. Therefore, we selected some modules (carboxylation module, decarboxylation module, malate transporter module, and stomatal regulation module) to generate CBP lines that only increase malate levels by up to 31%. This suggests that the malate content in synthetic materials still needs improvement. There may be other modules involved, such as the malate redistribution module, anatomical module, etc., which should be further integrated into this pathway in the future.

The CAM bypass we have created is only a conceptual product, but it still has the following shortcomings: 1) Stomatal opening is important for C_3_ plants, and long‐term open stomata are beneficial for CO_2_ entry into mesophyll cells and carbon sequestration. However, this also accelerates the leaf water evaporation. This can be sustained in aquatic plants, but it can be fatal in drought‐prone plants such as wheat. Drought or high‐temperature‐induced promoters can be used to drive the expression of CAM photosynthetic genes to avoid these problems. 2) CAM and C_3_ species show not only different biochemical processes but also different anatomical configurations. For example, leaf anatomical configurations, featuring enlarged and densely packed palisade mesophyll cells (indicative of succulence), as well as large vacuoles, can accommodate higher nocturnal C_4_ carboxylation and organic acid storage capacities. 3) There are possible metabolic pathways into the TCA cycle via oxaloacetate and malate. Targeted metabolic profiling shows that the CBP pathway significantly increases the metabolic flux of the TCA cycle. Apart from changes in the levels of malate and oxaloacetate, other metabolites, such as citrate, isocitrate, succinate, ɑ‐ketoglutarate, and fumaric acid, showed varying degrees of change in the CBP‐1 line. The increase in TCA pathway intermediates may indicate that the introduction of the CBP pathway activates the TCA cycle, leading to a corresponding up‐regulation of respiratory activity to provide more energy to sustain CBP metabolism. Further optimizing the regulation of the TCA cycle is therefore essential to prevent wasteful respiration of C_4_ acid and ensure an efficient CBP metabolic pathway. The CAM metabolic cycle is dependent on enzymes and transporter proteins, and it requires strict regulatory control to avoid futile cycles between carboxylation and decarboxylation.

In conclusion, we used a multi‐transgene stacking system to integrate CAM functional modules into C_3_ rice, synthesizing a novel shuttle that enables CAM photosynthesis. This opens up the possibility of implementing CAM engineering in C_3_ rice.

## 4 Experimental Section

### Cloning of the Target Genes and Subcloning into the Entry Vectors

The CAM core photosynthetic cycle modules included AcoMDH1 (Aco013935), AcoPEPCK1 (XM_020232666.1), AcoPPDK1 (Aco024818), AcoCA1(Aco006181), AcoPEPC1 (Aco010025) and their proteins were optimized and synthesized; AtALMT9 (AT3G18440) was amplified by PCR in *Arabidopsis* thaliana ecotype Columbia.^[^ ^33^ ^]^ AcoPEPCK1 and AcoPEPC11 were amplified based on the synthesized template using specific primers flanked by specific attB sites (using PEPCK1‐F/PEPCK1‐R; PEPC1‐F/PEPC1‐R primers), respectively, and products attB1 and attB3‐flanked product attB‐AcoPEPCK1, attB‐AcoPEPC1. A BP recombination reaction was performed between the attB‐AcoPEPCK1 or attB‐AcoPEPC1 product and the donor vector pGN2103CAK to produce the pGN2103CA‐AcoPEPCK1 or pGN2103CA‐AcoPEPC1 sets, respectively.

AcoMDH1 was amplified using the MDH1‐F/MDH1‐R primer and inserted into the *NotI* site of pGN2103CA‐AcoPEPCK1 to generate the pGN2103CA ‐AcoPEPCK1‐AcoMDH1 (entry 1). AcoCA1 was amplified using the CA1‐F/CA1‐R primer and inserted into the *NotI* site of pGN2103CA‐AcoPEPC1 to generate the pGN2103CA‐AcoPEPC1‐AcoCA1 (entry 3). AcoPPDK1 was amplified using a specific primer flanked by a specific attB site (use PPDK1‐F/PPDK1‐R primer) to produce attB3 and attB1‐flanked products, attB‐AcoPPDK1. A BP recombination reaction was performed between the attB‐AcoPPDK1 product and the donor vector pGN2104CGK to construct pGN2104CG‐AcoPPDK1 (entry 2). AtALMT9 was amplified under the control of the OsActin1 promoter using the AtALMT9‐F/AtALMT9‐R primer and then inserted into the Hind III site of L3L4‐NPT II vector set to construct the L3L4‐ NPT II‐AtALMT9 construct (entry 4). Details of all the primers used are given in Table S7 (Supporting Information).

### Multi‐Gene Assembly

We used the LR reaction‐mediated gene stacking system.^[^ ^34^ ^]^ For the stacking reaction, Gateway LR Clonase II Enzyme mix (Invitrogen, 11791020)‐mediated LR recombination was used.

In round 1, the entry1 construct for the round 1 LR reaction carries AcoMDH1 and AcoPEPCK1 expression cassettes adjacent to AmpR gene and sacB gene, which confers sensitivity to sucrose. The outermost attL1/attL2 sites recombined with attR1/attR2 on the destination vector, and the integrated attR3/attR4 sites on the intermediate destination were available for the next round of LR reaction.

In round 2, the entry2 construct for the round 2 LR reaction carried the AcoPPDK1 expression cassette, and attR1/attR2 carried by entry2 were integrated into the intermediate destination of round 1, generating a new intermediate destination selected with kanamycin and gentamicin.

In round 3, the entry3 for the round 3 LR reaction carries the AcoCA1 and AcoPEPC1 expression cassettes, and the attR3/attR4 sites carried by entry3 were integrated into the intermediate destination of round 2, generating a new intermediate destination selected using kanamycin and ampicillin.

In round 4, the stacking was completed by entry 4 carrying the NPT II and AtALMT9 expression cassettes, and the target recombinant was selected by sucrose to obtain the final pCBP construct.

### OsSLAC1 Knockout Construct

OsSLAC1 knockout construct was performed using the CRISPR‐Cas9 tool.^[^ ^35^ ^]^ A target sequence of OsSLAC1 (TTCAGCCGGCAGGTCTCCCT) was designed, then by overlapping PCR (using OsU6cF/OsSLACR or OsSLACF/gRNAR primers), the above two sequences were assembled into single fragments flanked by two *BsaI* sites of pYLCRISPR‐Cas9 and to generate the pYLCRISPR‐Cas9‐OsSLAC1 construct. Table S7 (Supporting Information) provides details of all primers used.

### Rice Genetic Transformation

The CBP construct and pYLCRISPR‐Cas9‐OsSLAC1 were introduced into A. *tumefaciens* strain AGL1 by electroporation. First, the pYLCRISPRCas9‐OsSLAC1 construct was used to infect *Nipponbare* calli by *Agrobacterium*‐mediated transformation. The SLAC1 knockout mutants were identified by PCR sequencing (using primers C‐SLAC1F/C‐SLAC1R). Second, the CBP construct was used to infect the *slac1* callus using the *Agrobacterium*‐mediated transformation method. The regenerated plants were analyzed by PCR using gene‐specific primers to detect the absence or presence of the six target genes.

### Subcellular Localization

The full‐length cDNAs of AcoMDH1, AcoPEPCK1, AcoPPDK1, AcoCA1, AcoPEPC1, AtALMT9, were cloned into the pAN580 to express the AcoMDH1‐GFP, AcoPEPC1‐GFP, AcoPEPCK1‐GFP, AcoPPDK1‐GFP, AcoCA1‐GFP, AtALMT9‐GFP fusion protein at the N terminus of GFP, respectively. The fusion construct and control construct of the cytoplasmic marker OsSWEET11 (P35S::OsSWEET11‐mRFP) were transformed into rice protoplasts. The transient transformation of these genes was performed by extracting leaf protoplasts from 2‐week‐old *Nipponbare* seedlings and using the polyethylene glycol method. After 14 h, the cells were examined and taken photos using laser scanning confocal microscopy (TCS SP2, Leica). The primers used are listed in Table S7 (Supporting Information).

### Real‐Time qRT‐PCR and RT‐PCR Analysis

Rice was grown in a greenhouse with day/night temperature of 28 °C/22 °C and a diurnal light regime of 14 h of light (white light‐emitting diodes with light density 350 µmol m^−2^ s^−1^) and 10 h of darkness. Leaf samples were harvested every 4 h, with a cycle of 24 h from the youngest fully expanded leaf of plants at the tiller stage, and immediately frozen. For expression analysis, total RNA was extracted from the samples using TRIzol reagent (Invitrogen) and reverse transcribed according to the manufacturer's instructions (TIANGEN). The rice ACTIN1 gene (Os03g0718100) was used as an internal standard to normalize the expression levels of genes such as *AcoMDH1, AcoPEPCK1, AcoPPDK1, AcoCA1, AcoPEPC1*. RT‐qPCR was performed using an iQ5 real‐time PCR detection system (Bio‐Rad) with real‐time PCR Master Mix (Vazyme, China). Primers used for expression analysis are described in Table S7 (Supporting Information). All experiments were performed with 3 biological replicates and 3 technical replicates (three amplified tubes for each example at each PCR assay time).

### Immunoblotting Assay

Rice leaves (100 mg) were frozen in liquid nitrogen, grounded, and then centrifuged at 12000 rpm for 20 min at 4 °C after adding protein extraction buffer (50 mm potassium phosphate [pH 7.5], 0.1% [v/v] Triton‐X 100, 1 mm EDTA, 5 mm MgCl_2_) to remove cellular debris. Using the Plant Nucleus and Cytoplasm Separation Kit (BB‐361124‐50T), we extracted cytoplasmic proteins from the leaves of both CK and CBP plants. Subsequently, chloroplast proteins were isolated and detected from these leaves, following previously reported protocols.^[^ ^36^ ^]^

SDS‐PAGE was performed using 10 µL of extracted leaf proteins. Antibodies, including mouse polyclonal anti‐AcoPEPC1, anti‐AcoPPDK1, anti‐AcoPEPCK1, anti‐AcoMDH1, anti‐AcoCA1, and anti‐AtALMT9 antibodies, were sourced from Abclonal in China.

### Enzyme Activity Measurement

Leaf samples (100 mg) were harvested at dawn (6:00 am) from flag leaves at the tillering stage, and immediately frozen. Leaf samples were homogenized to a fine powder using a nitrogen‐cooled mortar and pestle and extracted in 700 µL of buffer containing: 50 mm HEPES‐KOH, pH 7.5, 1 mm EDTA, 1 mm dithiothreitol, 5 mm MgCl_2_, 1% (v/v) glycerol. After centrifugation at 10000×g for 2 min at 4 °C, the supernatant was collected for enzyme activity measurements. For the AcoPEPCK assay, leaf samples were extracted in 50 mm HEPES‐KOH, pH 7.5, 5 mm DTT, 2 mm EDTA, 2 mM MnCl_2_, 1% (w/v) PVPP and 0.05% Triton. No PEPC background activity was observed when MgCl_2_ was omitted from the extraction and assay buffer.

The PEPC reaction mixture contains 100 mM HEPES‐KOH, pH 7.5, 10 mm MgCl_2_, 0.2 mm NADH, 1 mm NaHCO_3_, 5 mm G6P, 8ug MDH (from pig heart; Roche Diagnostics Gmbh) and 4 mm PEP. The reaction was started by adding to leaf extract.

PPDK reaction mixture contained 50 mm HEPES‐KOH, pH 7.5, 0.1 mm EDTA, 3 mm DTT, 10 mm MgCl_2_, 10 mm NaHCO_3_, 1.25 mm pyruvate, 0.2 mm NADH, 8ug MDH (from pig heart; Roche Diagnostics Diagnostics Gmbh), 2.5 mm KH2PO4, 8ug PEPC (S10210‐100U) and 1.25 mm ATP. The reaction was initiated by the addition to leaf extract.

NADP‐MDH reaction mixture contains 50 mm HEPES‐KOH, pH 7.5, 70 mm KCl, 1 mm EDTA, 1 mm DTT, 0.2 mm NADPH, and 2 mm OAA. The reaction was initiated by the addition to leaf extract.

PEPCK reaction mixture contains 50 mm HEPES‐KOH, pH 7.5, 15 mm PEP 2 mm DTT, 2 mm MnCl_2_, 90 mm NaHCO_3_, 100 mm KCL, 0.14 mm NADH, 8ug MDH (from pig heart; Roche Diagnostics, Basel, Switzerland) and 1 mm ADP. The reaction was initiated by the addition to leaf extract.

The activities of PEPC, PPDK, NADP‐MDH, and PEPCK enzymes were measured spectrophotometrically at 340 nm at 25 °C, respectively.

### Titratable Acidity Assay

The CBP lines and CK leaf tissues were collected in greenhouses at 12:00 pm (day) and 12:00 am (night). The Aloe vera green tissues were collected at dawn and dusk. Titratable acidity was measured by grinding ≈0.2 g of leaf tissue in liquid nitrogen using a mortar and pestle. Malate content was determined using the Solarbio (China) Titratable Acid (TA) Detection Kit (via titration method) according to the kit instructions.

### Organic Acids HPLC‐MS Determination

The CBP lines and CK leaf tissues were collected at 12:00 pm (day) and 12:00 am (night). HPLC/MS determination of organic acids was measured by grinding ≈100 mg of leaf tissue in liquid nitrogen using a mortar and pestle, adding 3 mL of extraction solution (water, methanol, and chloroform in the ratio of 1:2.5:1); then adding 1 ppm of 2‐aminobutyric acid as an internal standard. The sample is vortexed for 20 s, sonicated in ice water for 30 min, then centrifuged at 12000 × g for 5 min to obtain the supernatant, which is stored at −80 °C for further processing. Add 100 µL of water to 200 µL of supernatant and centrifuge at 12000 × g for 5 min. Transfer 150 µL of the new supernatant to insert tube of the HPLC vials and load into the Agilent 498 Multisampler (G7167B). The liquid chromatography and mass spectrometry conditions were as follows: for organic acids: a Hypersil GOLD aQ column (2.1 × 100 mm, 1.9 µm particle size, ThermoFisher Scientific) was used, and the mobile phase system consisted of 0.3% aqueous formic acid aqueous solution (A) and 100% methanol (B), with the temperature maintained at 35 °C. The injection volume was 5.0 µL. The mass spectrometry conditions were such that MS/MS data were acquired in negative MRM mode. The capillary voltage was set to 3.5 kV, and nitrogen gas was used for nebulization (35 psig), drying (11 L min^−1^, 150 °C), and sheath gas (11 L min^−1^, 150 °C). The residence time and fragmentation voltage were 40 ms and 125 V, respectively. LC‐MS data were analyzed and quantified using Agilent's MassHunter Qualitative Navigator and QQQ Quantitative Analysis software from. For relative quantification, the peak areas of the metabolites were normalized to the internal extraction standard and to the fresh weight of the material.

### Photosynthesis Measurement

The CBP lines and the CK were planted in the field under normal growth conditions. At the flowering stage, gas exchange measurements, CO_2_ response curves, and light response curves were measured using a portable photosynthesis system (LICOR6800, Licor Bioscience). CO_2_ response curves were obtained at a leaf temperature of 30 °C, 60% relative humidity, and 1000 µmol m^−2^s^−1^ PFD; A/Ci response curves were recorded at different CO_2_ levels, 400, 300, 200, 150, 100, 80, 70, 60, 50, 400, 400, 400, 600, 800, 1000, 1500 µmol mol^−1^. The initial slope of the CO_2_ response curve was calculated in the linear range between 0 and 200 µbar external CO_2_, and the CO_2_ compensation point (Г) and maximum CE were calculated from the intercept and slope of the CO_2_ response curves.

Light‐response curves were obtained at a leaf temperature of 30 °C, 60% relative humidity and CO_2_ concentration of 400 µmol mol^−1^; PFD was gradually reduced from 2000,1500,1000500300200150120,90,60,40,20,0 µmol m^−2^ s^−1^.

Quantum yield for CO_2_ assimilation(ΦCO_2_) and R_d_ was calculated from the slope and intercept of the light response curves (PPFD <100 µmol photons m^−2^s^−1^). A_max_ was calculated from the light response curves. The values of A and gsw were used to estimate iWUE (iWUE = A/gsw).

The Fo (minimum fluorescence) and Fm (maximal fluorescence) of leaves in the dark for 15 min were measured. After actinic light and saturation pulse values were applied, leaf chlorophyll fluorescence parameters, Fv/Fm (maximum efficiency of PSII photochemistry under dark‐adaption).

### Targeted Metabolite Analysis

At the flowering stage, leaves of CK and CBP1 plants were collected for metabolomics assay. To ensure the accuracy of our analysis, we collected one replicate of the middle part of the rice flag leaf, and three biological replicates were collected at dawn (6:00) and dusk (18:00). The analysis and testing of the samples were completed by Biomarker Biotech Company.

For metabolite profiling, samples were harvested by immediate quenching with liquid nitrogen. An appropriate amount of sample(≈20 mg FW) was weighted into a 2 mL centrifuge tube, steel balls were added and 1 mL acetonitrile: water solution (7:3, V/V, containing standard mixture) was vortexed for 30 s; 25 Hz homogenized for 10 min, then vortexed for 3 min, placed on ice and extracted for 30 min; samples were centrifuged at 4 °C at 12 000 r min^−1^ for 10 min; the supernatant was collected at 500 µL over 0.22 µm membrane injection analysis. The UHPLC separation was performed using a Waters ACQUITY I‐Class, equipped with an ACQUITY UPLC BEH Amide 1.7 µm (1.7 µm 2.1*150 mm, waters). The mobile phase A2: water: acetonitrile = 95:5 (containing 0.05% ammonia and 5 µm methylene diphosphate); B1: water: acetonitrile = 5:95 (containing 0.05% ammonia and 5 µm methylene diphosphate). Column temperature was set at 40 °C. The autosampler temperature was set at 10 °C and the injection volume was 1 µL. A SCIEX 6500 QTRAP+ triple quadrupole mass spectrometer (Sciex), equipped with an IonDrive Turbo V electrospray ionization (ESI) interface, was used for assay development. The MRM parameters for each of the target analytes were optimized using flow injection analysis by injecting the standard solutions of the individual analytes into the API source of the mass spectrometer. Several most sensitive transitions were used in the MRM scan mode to optimize the collision energy for each Q1/Q3 pair. From the optimized MRM transitions per analyte, the Q1/Q3 pairs with the highest sensitivity and selectivity were selected as “quantifier” for quantitative monitoring. The additional transitions were used as “qualifiers” to verify the identity of the target analytes. SCIEX Analyst Work Station software (version 1.7.2) and Sciex OS 2.0.1 were used for MRM data acquisition and processing.

### Drought Treatments

Simulation of drought stress experiments by polyethylene glycol (PEG) was carried out in a growth chamber. Thirty seeds each from the CK and CBP lines (an independent transformant) at the T_2_ generation were placed in a 10‐cm Petri dish with 20 mL of sterile distilled water. All were germinated for ≈3 days in a 28 °C incubator, transferred to 96‐well plastic plates with nutrient solution, and grown to the fifth leaf stage. Next, the nutrient solution was replaced by a 20% PEG 4000 solution. After all the plants wilted, the PEG solution was replaced with a nutrient solution. After ≈7 days, images were acquired. Seedling survival rates were recorded.

To judge the performance of CK and CBP lines in response to drought stress, we grew the plants in pots (50 cm × 20 cm × 15 cm) containing commercial peat soil in the growth chamber. The plants were irrigated with no interval. Natural drought stress treatments were carried out at the later tillering stage until the plants wilted completely and were then rehydrated for several days.

### Measurement of Vacuolar pH

Vacuolar pH was monitored with the cell‐permeant and pH‐sensitive fluorescent dye BCECF‐AM in rice protoplasts. Extract leaf protoplasts from 2‐week‐old *Nipponbare* seedlings at night (12:00 am). Then, freshly prepared protoplasts were loaded with the fluorescent dye in MMG solution (0.6 m mannitol, 15 mm MgCl2, 4 mm MES, pH 5.7) supplemented with 10 µm BCECF‐AM; After 1 h staining at 28 °C in the darkness, the protoplasts were washed twice with W5 solution (154 mm NaCl, 125 mm CaCl_2_, 5 mm KCl, 2 mm MES, pH 5.7), and then incubated in W5.

For in situ calibration of BCECF, the protoplasts were incubated in serial pH equilibration buffers containing 0.4 m mannitol, 20 mm KCl, 50 mm ammonium acetate supplemented with 50 mm MES (pH 5.2‐6.4) or 50 mm HEPES (pH 6.8–7.6), respectively.

The BCECF fluorescence was detected and imaged using laser scanning confocal microscopy (TCS SP2, Leica). The fluorophore was excited at 488 and 458 nm, respectively, and the emission was detected between 505 and 550 nm. Vacuolar pH value was quantified by ratio analysis of the pH‐dependent (488 nm) and pH‐independent (458 nm) excitation wavelengths from a calibration curve (Figure S8, Supporting Information), and ratio images were produced using the ion concentration tool of Leica software.

### Primer Sequences

The primers used are described in Table S7 (Supporting Information).

### Quantification and Statistical Analysis

Statistical analysis for all experiments was performed in Graphpad Prism 8 using one‐way analysis of variance (ANOVA)and Tukey post hoc test or Student's t‐test with a *p*‐value < 0.05.

## Conflict of Interest

The authors declare no conflicts of interest.

## Author Contributions

W.S.T. conceived the main experiments. J.K.N. conducted the photosynthetic measurement. Z.S.S. conducted the vector construction. L.Y.N. performed the HPLC /MS measurements. L.H.S and Y.J.W. performed the transformation experiments. Z.S.S. carried out the expression analysis. Z.L.Y. went on the subcellular localization. Y.J.W went on the field management. S.X.H, C.G.X, C.X.A, and S.J. contributed to the overall data discussion. W.S.T and Z.Z.G. contributed to the overall data analysis. Z.Z.G and L.T.G. designed and supervised the writing of the manuscript.

## Supporting information



Supporting Information

## Data Availability

The data that support the findings of this study are available from the corresponding author upon reasonable request.
