# Supplementary material for: A Synthetic Facultative CAM‐Like Shuttle in C3 Rice Plants
Source: Adv Sci (Weinh). 2025 Feb 7;12(13):2500418. doi: 10.1002/advs.202500418 (PMC11967806; doi:10.1002/advs.202500418)
Supplement: Supplementary file 1 — Supporting Information [file ADVS-12-2500418-s001.pdf]

ADVANCED SCIENCE
Open Access

Supporting Information

for *Adv. Sci.*, DOI 10.1002/advs.202500418

A Synthetic Facultative CAM-Like Shuttle in C_3 Rice Plants

Suting Wu, Kaining Jin, Haoshu Li, Guoxin Chen, Liying Zhang, Jinwen Yang, Shanshan Zhai, Yanni Li, Xuehui Sun, Xuean Cui, Jing Sun, Tiegang Lu and Zhiguo Zhang**

Supporting Information

A synthetic facultative CAM-like shuttle in C_3 rice plants

Suting Wu[1], Kaining Jin[1]*, Haoshu Li[1], Guoxin Chen[1], Liying Zhang[1], Jinwen Yang[1], Shanshan Zhai[1], Yanni Li[1], Xuehui Sun[1]. Xuean Cui[1], Jing Sun[1], Tiegang Lu[1#], Zhiguo Zhang[1#]*

* These authors contributed equally to this article.

# Authors for correspondence: lutiegang@caas.cn; zhangzhiguo@caas.cn.

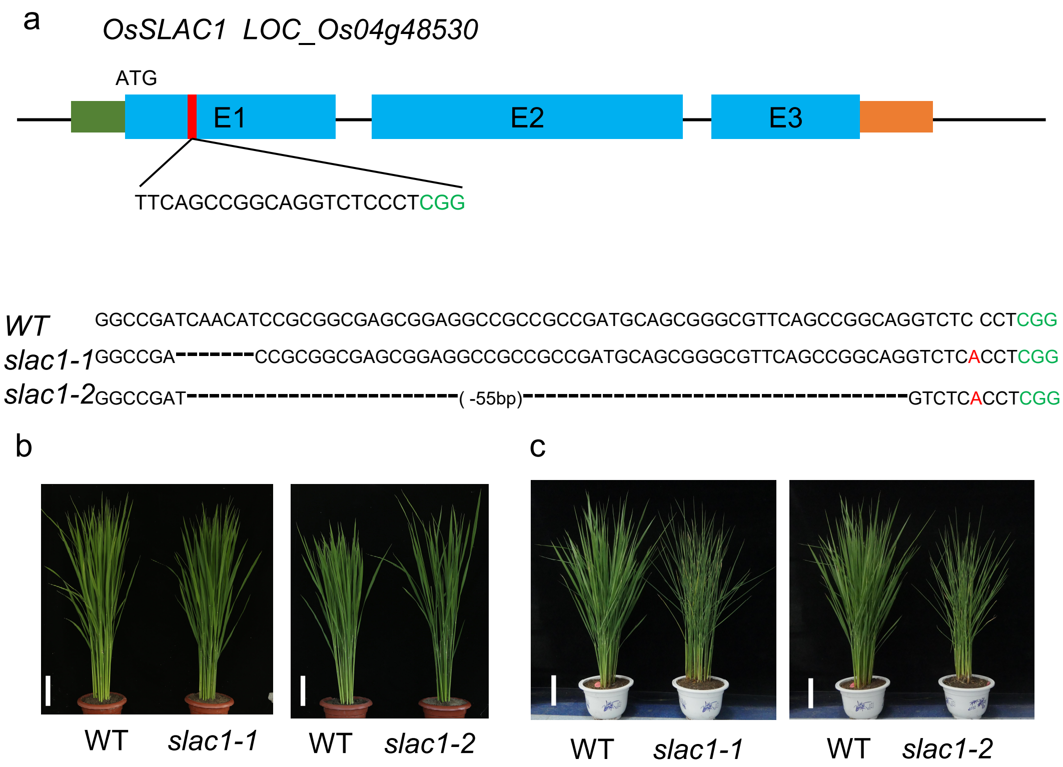

Fig. S1. Knockout SLAC1 using a gene-editing tool.

(a)The gene-specific spacer sequence was selected in the first exon of OsSLAC1; Chromatograms of the OsSLAC1 sequence in wild-type, *slac1-1* and *slac1-2*. Green bases show the protospacer adjacent motif (PAM) sequence, the dotted line means base deletions, red bases means base insertion. (b) Representative pictures of wild-type (WT) and two representative *slac1*. Scale bar, 10cm. (c) Mutants to dehydration display a rolled leaf phenotype. Scale bar, 10cm.

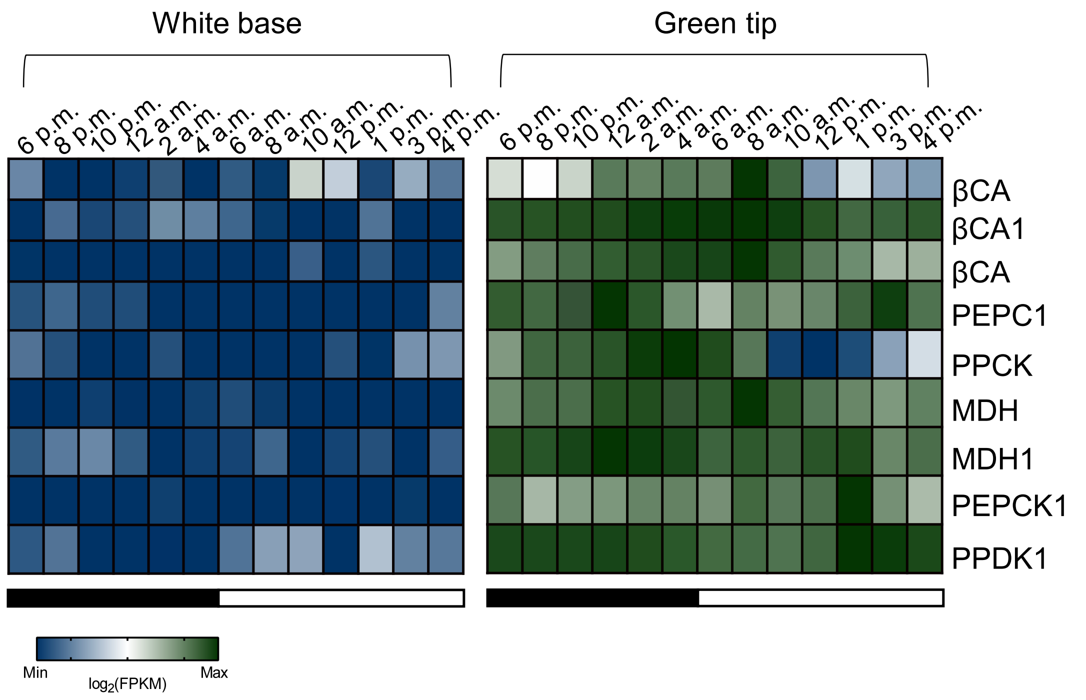

Fig. S2. Heatmap of carbon fixation genes Expression pattern in pineapple. Expression pattern of pineapple carbon fixation genes across the diurnal expression data. log_2-transformed fragments mapped per kilobase of transcript length per million total mapped reads (FPKM) expression profiles are shown (Ming et al., 2015).

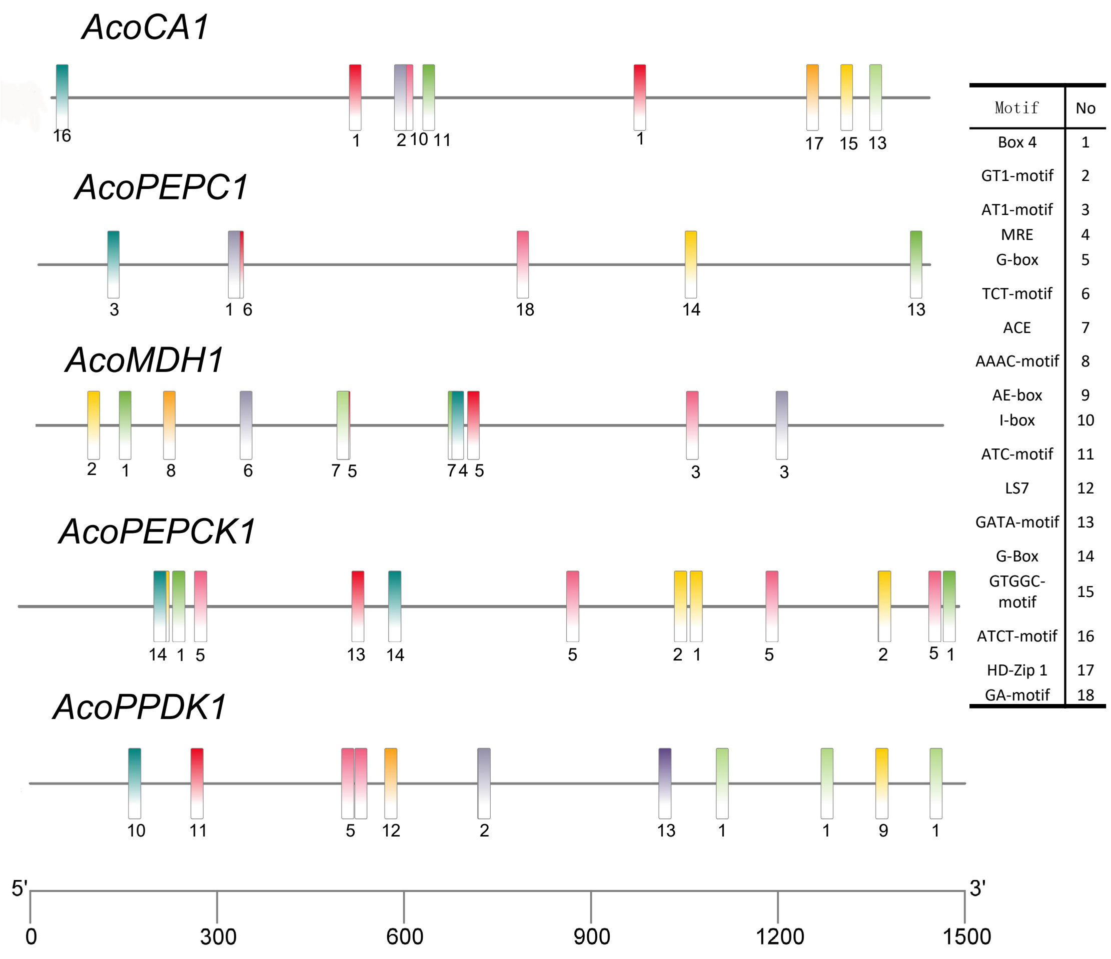

Fig. S3. The promoter of CAM core genes contains some of the elements involved in the regulation of light response.

The 1.5-kb upstream promoter sequences of pineapple genes were searched for five known circadian clock-related motif sequences. These motifs include the morning element (ME, CCACAC), evening element (EE, AAAATATCT), CCA1-binding site (AAAAATCT), G-box element (G-box, CACGTG), and TCP15-binding motif (TCP15, nGGnCCCAC).

PlantCARE: a database of plant cis-acting regulatory elements and a portal to tools for in silico analysis of promoter sequences.

https://bioinformatics.psb.ugent.be/webtools/plantcare/html/

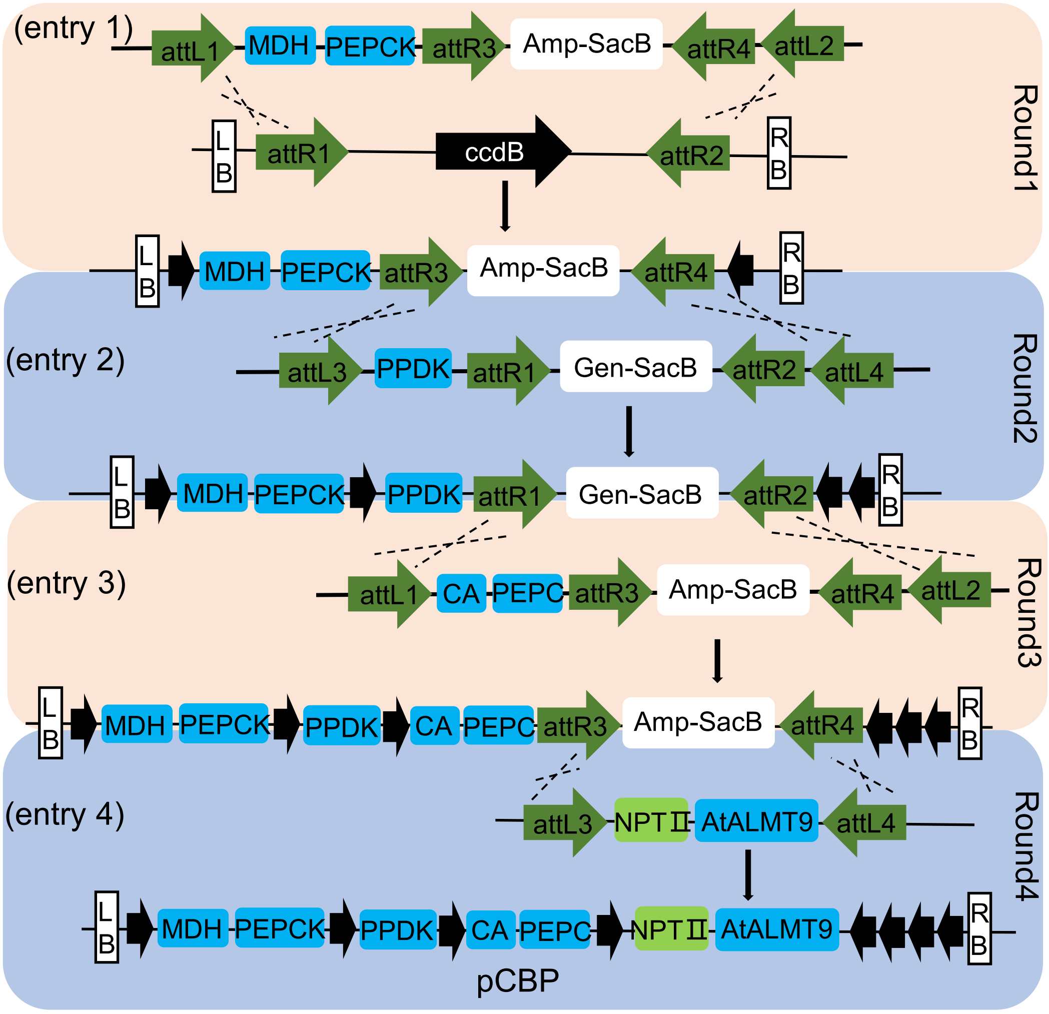

Fig. S4. Schematic diagram representation of the CBP multigene expression vectors using the GNS transgene stacking system.

In round 1, the entry1 vector for the round 1 LR reaction carries MDH and PEPCK expression cassettes, which are adjacent to the AmpR gene and the sacB gene conferring sensitivity to sucrose. The outermost attL1/attL2 sites can recombine with attR1/attR2 on the destination vector, and the integrated attR3/attR4 sites on the midestination are available for the next round of LR reaction. In round 2, the entry2 vector for the round 2 LR reaction carries the PPDK expression cassette, and attR1/attR2 carried by entry vector 2 are integrated into the midestination of round 1, generating a new midestination selected using kanamycin and gentamicin. In round 3, the entry3 vector for the round 3 LR reaction carries the CA and PEPC expression cassettes, and the attR3/attR4 sites carried by entry vector 3 are integrated into the

midestination of round 2, generating a new midestination selected using kanamycin and ampicillin. In round 4, stacking is completed by an entry 4 carrying the NPT Ⅱ and AtALMT9 expression cassettes, and the target recombinant is selected by sucrose to obtain the final expression clone, pCBP vector .

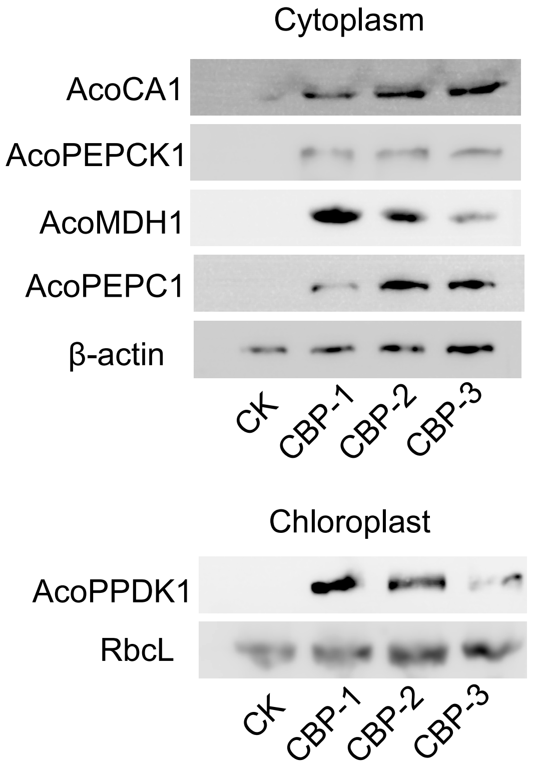

Fig.S5. Immunoblot analysis was performed on cytoplasm and chloroplast protein extracted from the leaves of CK and CBP plants. Rice β-actin or RbcL served as the control.

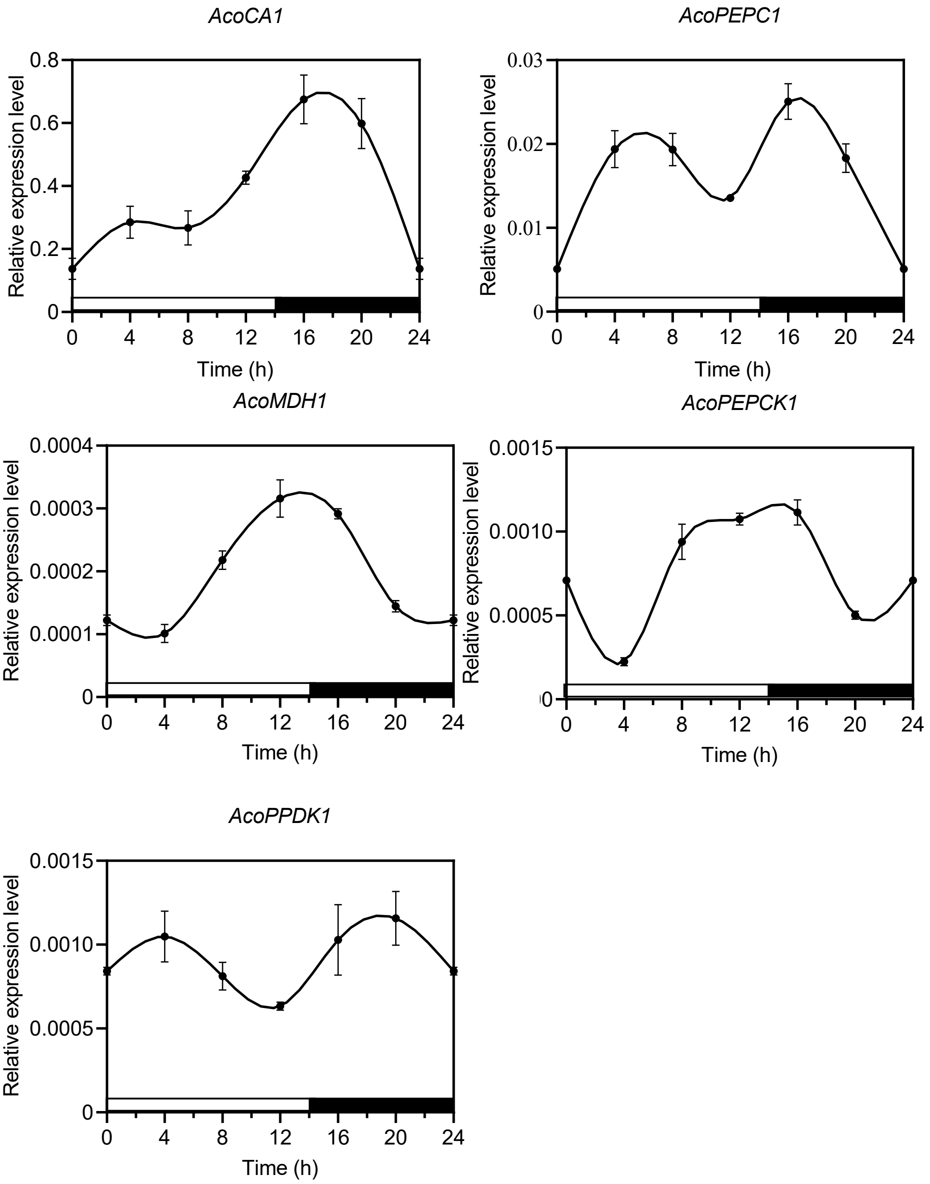

Fig. S6. Detection of circadian rhythm in CBP lines.

RT-qPCR analysis of the gene expression patterns of selected carboxylation and decarboxylation module genes in CBP lines. Samples are taken every 4 hours, with a cycle of 24 hours, during the alternation of day and night to obtain the diurnal expression map of five genes. The rice Actin gene was amplified as the internal control. The rice ubiquitin gene for quantitative analysis. The rice endogenous Actin gene (Os03g0718100) was selected as an internal reference to normalize the data. The abscissa is the lighting duration. n=3. Mean ± SD is presented.

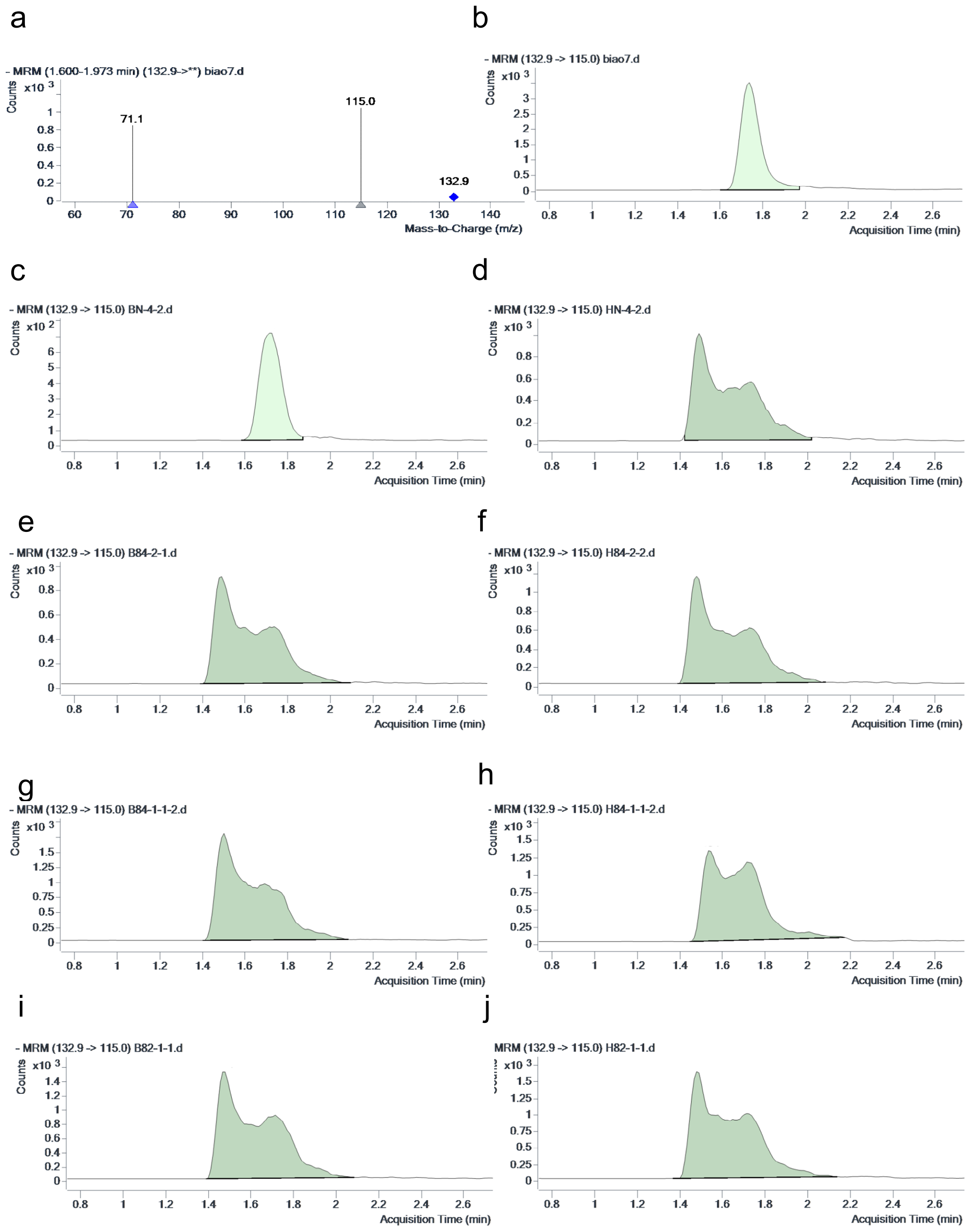

Fig. S7. HPLC/MS determination of malate. (a) Deconvoluted mass spectrum of malate. (b) Liquid chromatograms of the malic acid specific masses standard. (c) and (d) Liquid chromatograms of the malic acid specific masses: CK, 12:00 pm (day) and 12:00 am (night). (e) and (f) Liquid chromatograms of the malic acid specific masses: CBP, 12:00 pm (day) and 12:00 am (night). (g) and (h) Liquid chromatograms of the malic acid specific masses: CBP, 12:00 pm (day) and 12:00 am (night). (i) and (j) Liquid chromatograms of the malic acid specific masses: CBP, 12:00 pm (day) and 12:00 am (night).

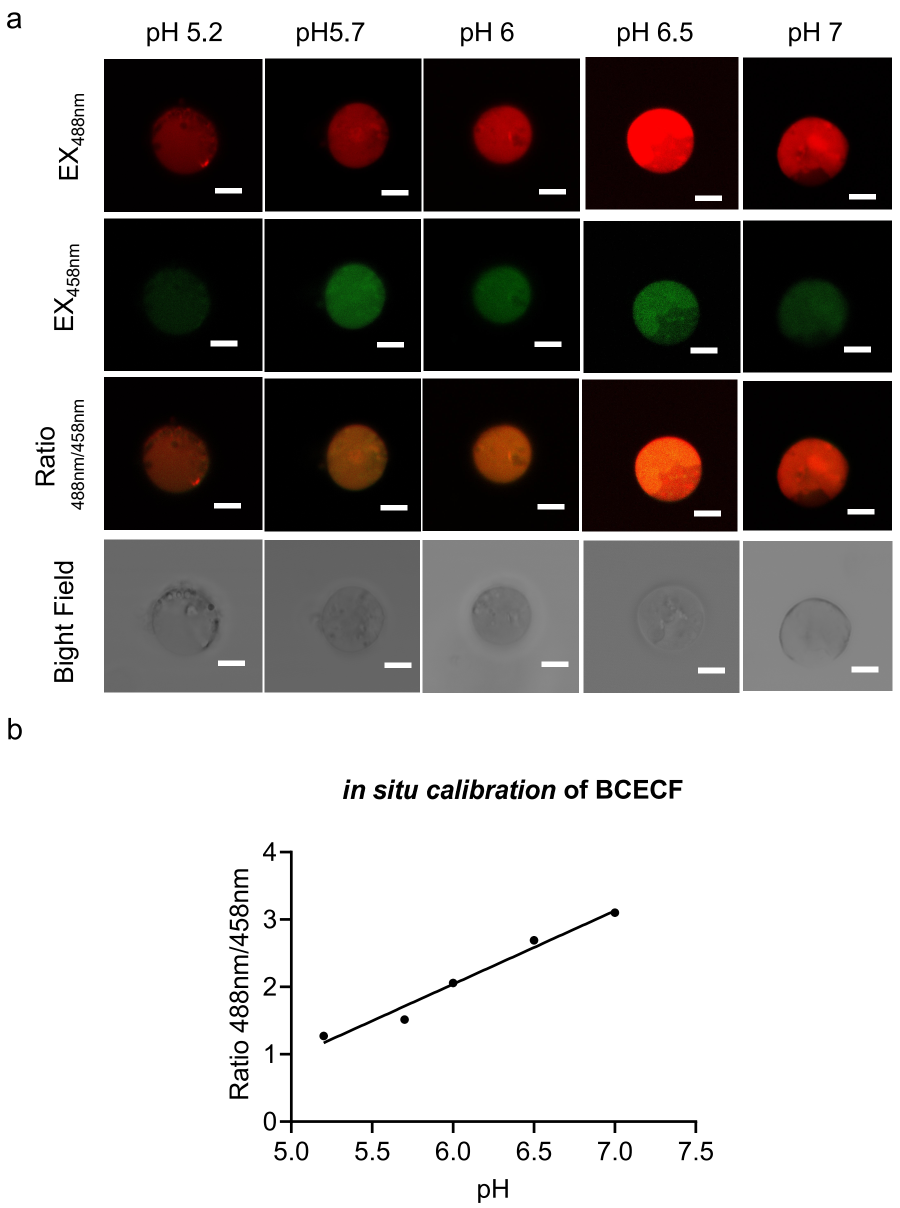

Fig. S8. In situ calibration of BCECF-AM in protoplast vacuoles.

(a) Protoplasts containing vacuolar BCECF in a series of equilibration buffers were imaged from 488 nm (panels in the top row, red) and 458 nm (panels in the second row, green) excitation of BCECF. Ratio images were pseudo-colored to show the pH gradient (panels in the third row). Bright field images were also captured from the same cell (panels in the bottom row). Scale bar=10 μm. (b) The fluorescence ratios (488/458 nm) were plotted against the pH value of the equilibration buffers to generate a calibration curve.

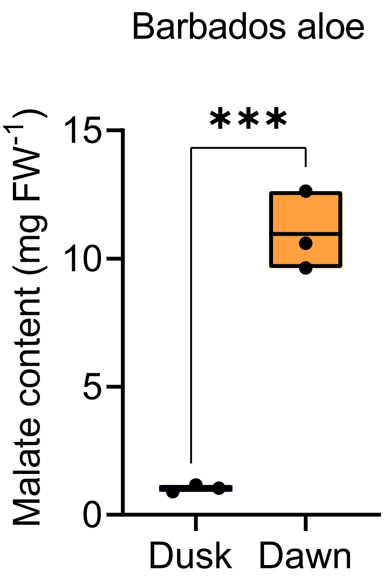

Fig. S9. Quantification of malate content in Aloe vera. Relative malate content per mg fresh weight (FW) of Titratable Acidity analysis of *Barbados aloe* at dawn 6:00 and dusk 18:00. n = 3. Asterisks represent significant differences according to Student's t test, ***$p < 0.001$.

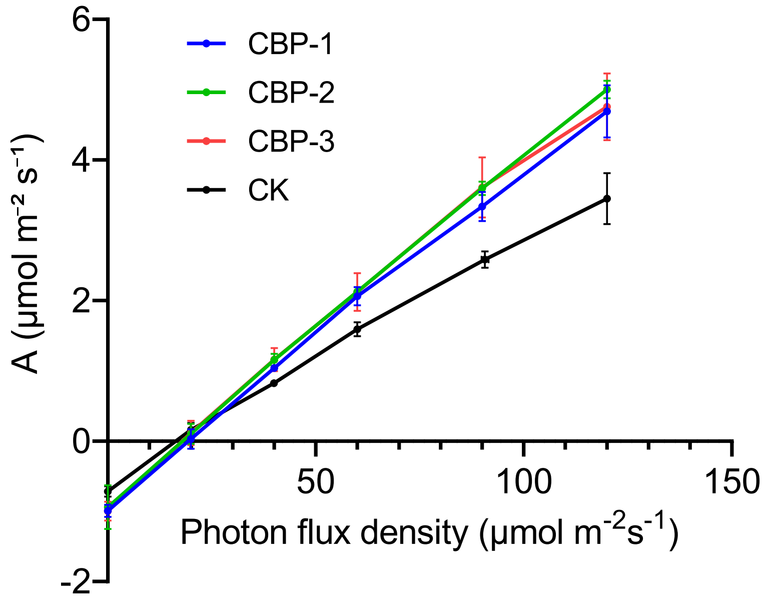

Fig. S10. Gas exchange measurements in low light.

Light response curves (the photon flux density in the range of 0~120 μmol $m^{-2}s^{-1}$) was generated at a temperature of 30 ℃ under normal air conditions and a CO_2 concentration of approximately 400 ppm. n = 3.

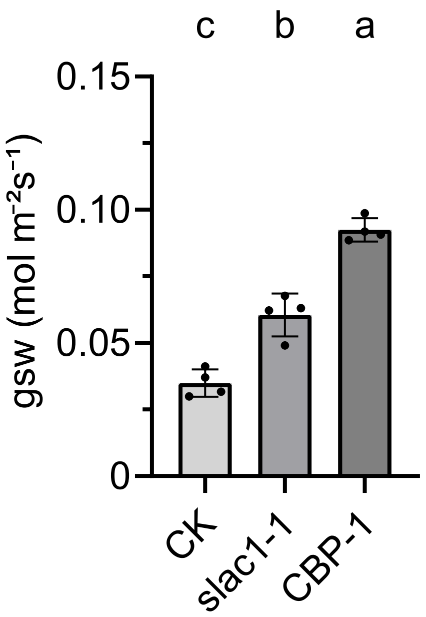

Fig. S11. Stomatal conductance (gsw) in three rice lines in the dark. Different letters (a–c) indicate significant differences ($P < 0.05$) between lines. n = 4.

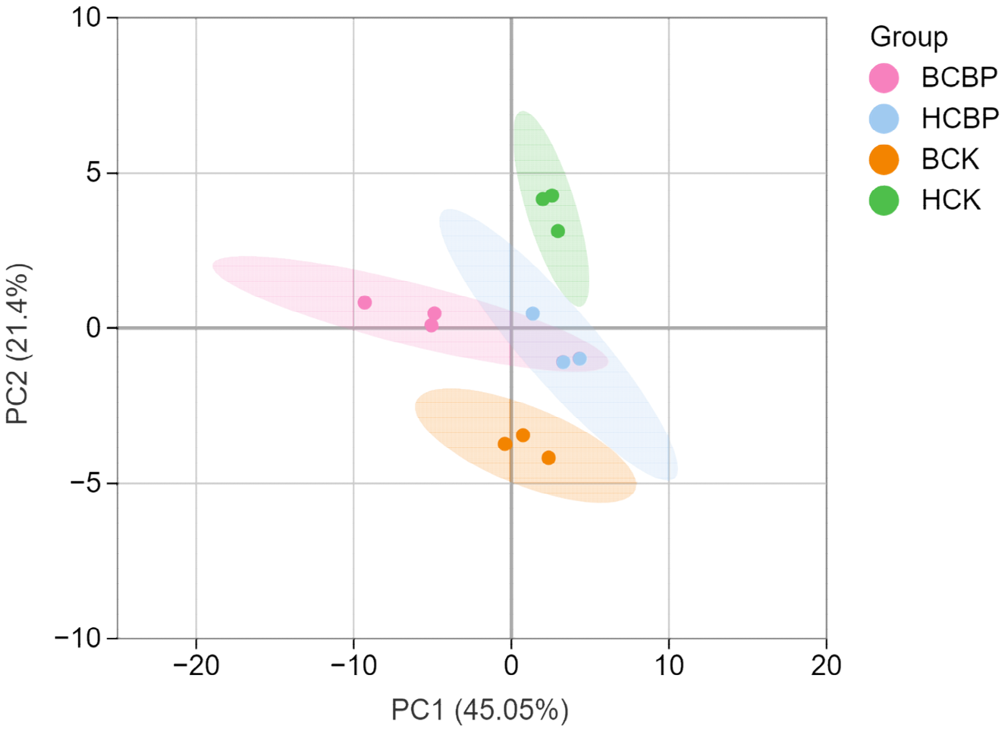

Fig. S12. Targeted metabolomics analysis of CK and CBP lines at 6:00 and 18:00 two points.

A principal component analysis (PCA) plot showing correlations among the samples between CK and CBP lines at different timepoints. BCBP represents the samples obtained at 6:00, HCBP represents the samples obtained at 18:00, BCK represents the samples obtained at 6:00, HCK represents the samples obtained at 18:00. n = 3.

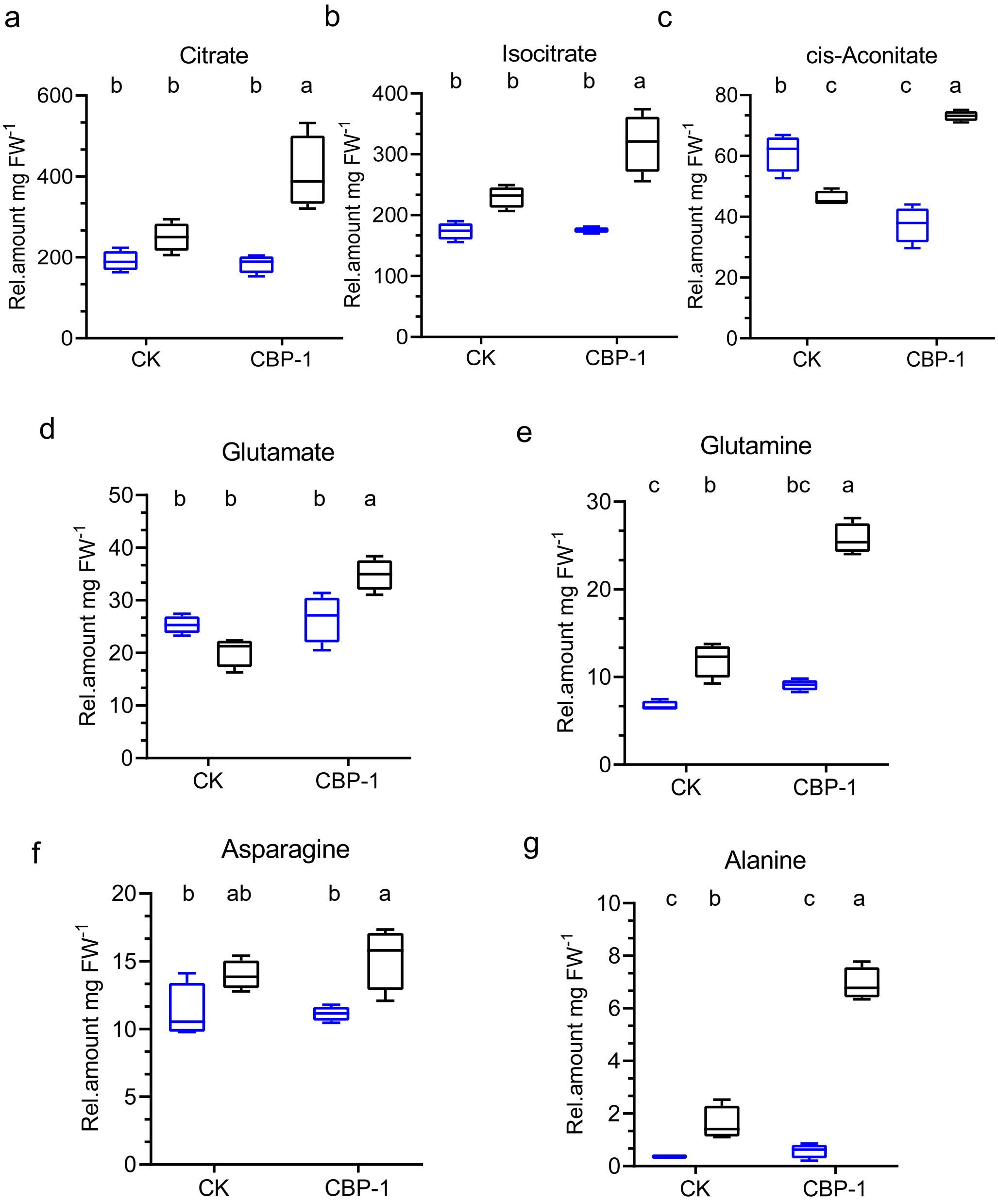

Fig. S13. Target metabolite contents in CK and CBP lines at 6:00 and 18:00 two points. Relative metabolite levels per mg FW (fresh weight) of (a) Citrate; (b) Isocitrate; (c) cis-Aconitate; (d) glutamate; (e) Glutamine; (f) Asparagine and (g) Alanine in CBP and CK lines. Black box represents the samples obtained at 6:00 and Blue represents the samples obtained at 18:00. n = 3. Bars (a) to (g) represent means ± SDs. Different letters (a–c) indicate significant differences ($P < 0.05$) between lines.

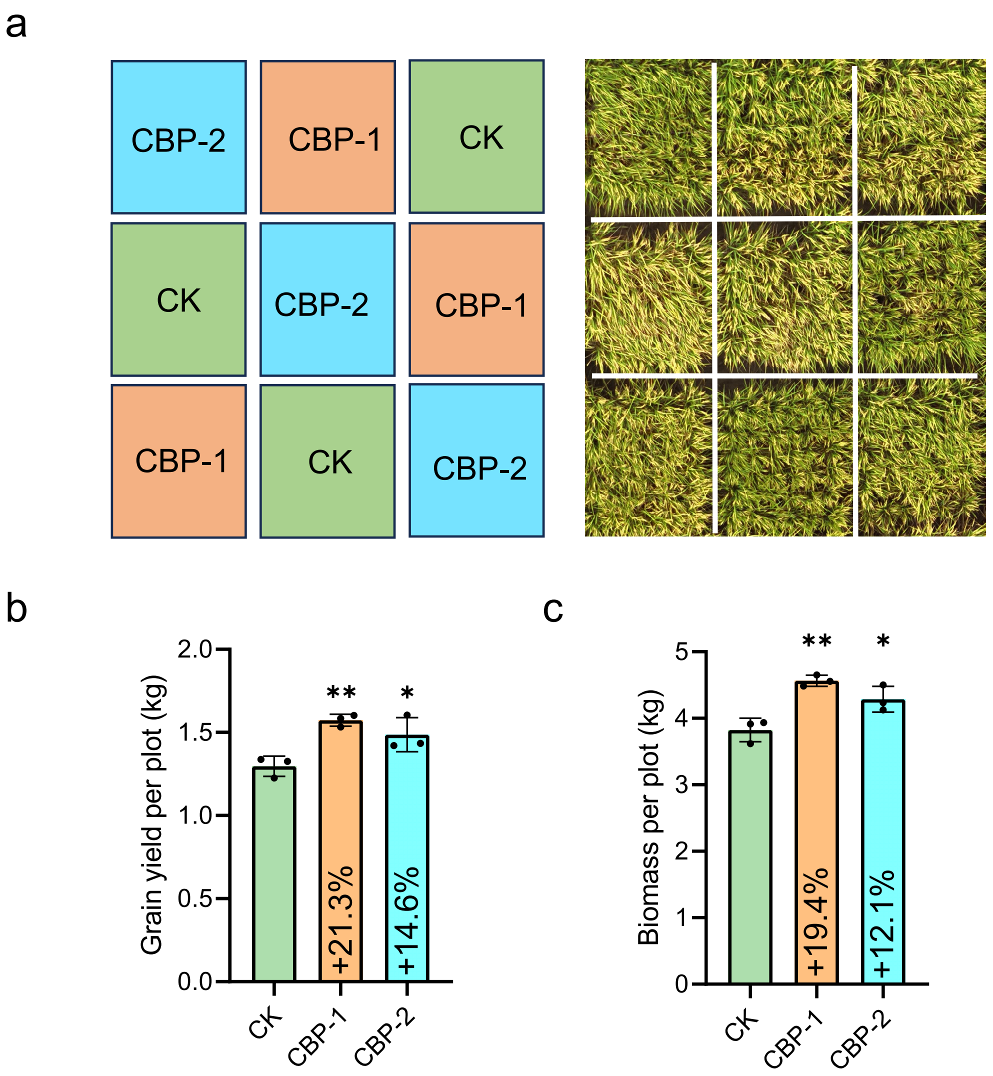

Fig. S14. Agronomic evaluation of CK and CBP lines.

(a) Photographs of CK and CBP plants in the field. A randomized plot design with three replications was used for all field experiments in this study. The entire experimental field was divided into three replicate blocks, each plot containing a 6×6 grid of plants. Nine plots were used for the three plant types, i.e., two independent CBP bypass transformation events (CBP-1 and CBP-2) and CK, with three plots for each type, respectively. Grain yield per plot (b) and biomass per plot (c) of CBP and CK in the field. Asterisks represent significant differences according to Student's t test, *, ** significance between the CBP lines and CK at $p < 0.05$, $p < 0.01$ respectively.

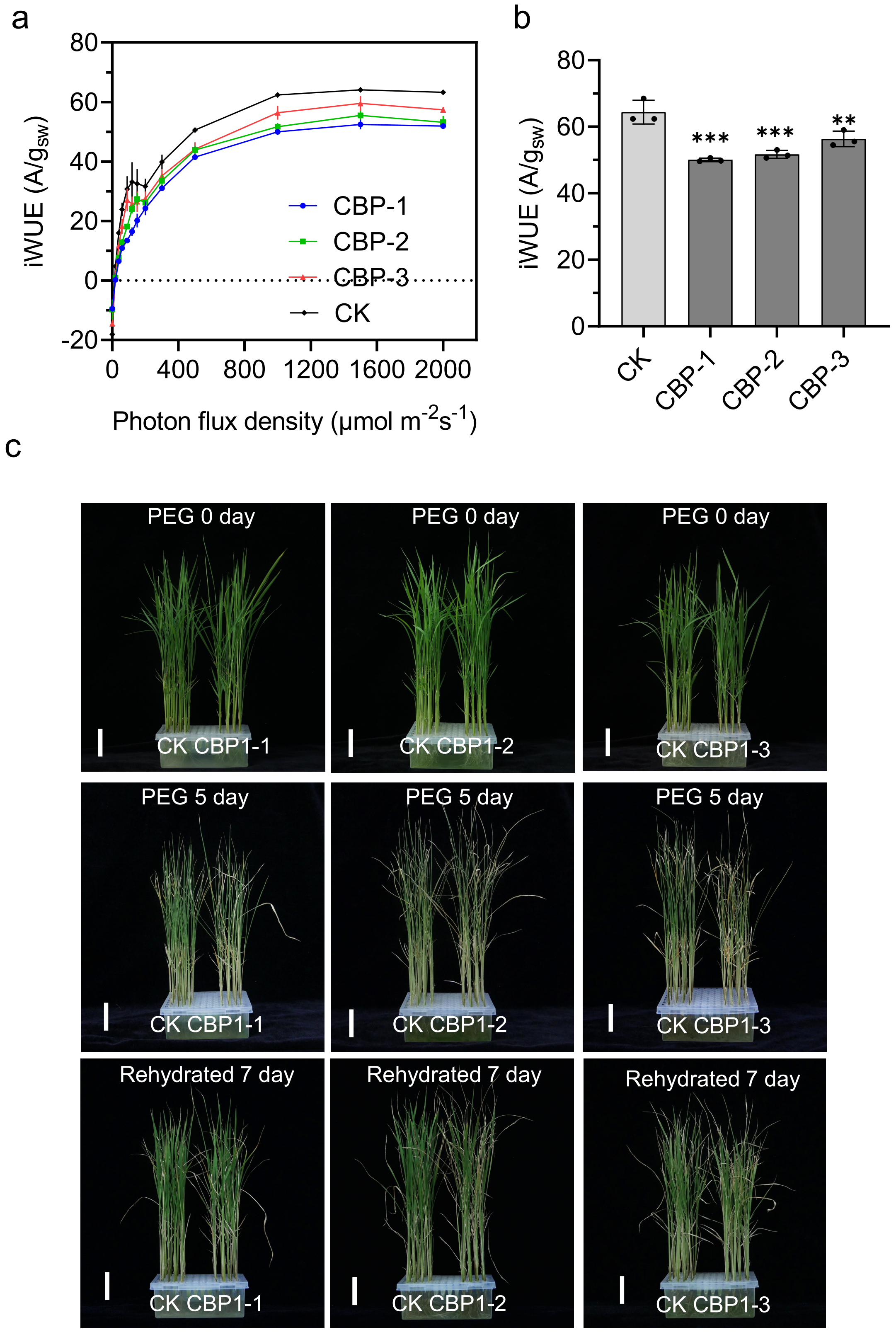

Fig. S15. Water use efficiency and tolerance to PEG-induced drought stress in the CK and CBP lines.

(a) Effects of light on the water-use efficiency in the CK and the CBP lines. The

values of A and g_{sw} were used to estimate iWUE (iWUE = A/g_{sw}).

(b) The iWUE was measured at photon flux density 1000 μmol $m^{-2}s^{-1}$, a temperature of 30℃, and a CO_2 concentration of approximately 400 ppm. All measurements were performed using the flag leaf of rice at the filling stage. Mean ± SD. n = 3; Asterisks represent significant differences according to Student's t test, $*p < 0.05$, $**p < 0.01$, $***p < 0.001$.

(c) Five-leaf stage CK and CBP1 seedlings grown in hydroponic culture solution then treated with 20% PEG reagent (0 day denotes before treatment), after 5 days of 20% PEG treatment and then rehydrated for 7 days. Scale bar, 5 cm.

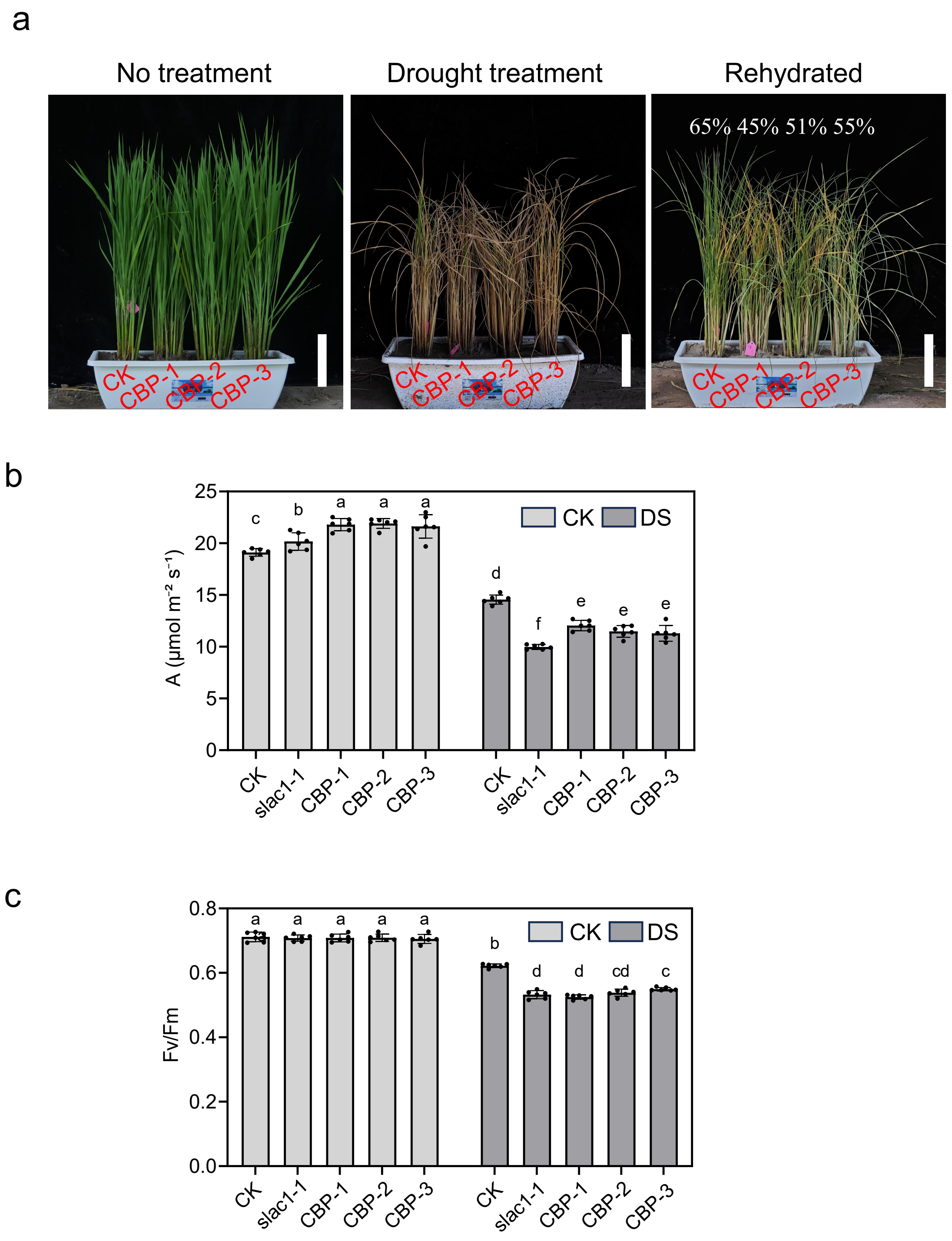

Fig. S16. Morphology and physiology in response to drought treatments in rice lines.

(a) The phenotype of transgenic rice under drought. Scale bar, 15 cm.

(b) photosynthesis rates A in three rice lines exposed to the drought stress (DS) treatment. Different letters (a–d) indicate significant differences ($P < 0.05$) between lines. n = 6.

(c) Maximum photochemical quantum yield (Fv/Fm) of PSII exposed to the drought stress (DS) treatment. Different letters (a–d) indicate significant differences ($P < 0.05$) between lines. n = 6.

Table S1. Genbank Number of selected genes in the CBP bypass.

Gene No.	Genebank No.	CDS length
AcoCA1	XM_020250721.1	1497bp
AcoPPDK1	XM_020231257.1	2868bp
AcoPEPC1	XM_020241176	2895bp
AcoPEPCK1	XM_020232666.1	2055bp
AcoMDH1	XM_020228970	999bp
AtALMT9	NM_112729.4	1794bp

Table S2. Numbers of putative carbon fixation genes in pineapple, orchid, rice and maize.

Gene	Pineapple	Orchid	Rice	Maize
Carbonic anhydrase (CA)	9	15	16	16
Phosphoenolpyruvate carboxylase (PEPC)	3	2	7	6
Phosphoenolpyruvate carboxylase-related kinase	2	2	2	2
Phosphoenolpyruvate carboxylase kinase (PEPCK)	1	1	2	3
Malate dehydrogenase (MDH)	14	5	9	13
Malic enzyme (ME)	5	5	7	8
Phosphoenolpyruvate carboxykinase (PPCK)	1	2	3	6
Pyruvate phosphate dikinase (PPDK)	1	1	2	2
Pyruvate phosphate dikinase regulatory protein	1	1	1	1

This overview summarizes information from previous reports on Pineapple CAM photosynthesis. (Ming et al., 2015).

Table S3. Enzyme activity analysis for CK and CBP lines

Enzyme activity ($U\ mg\ protein^{-1}$)	CK	CBP-1	CBP-2	CBP-3
PEPC	8.53 ± 0.95^{b}	12.91 ± 0.97^{a}	13.74 ± 0.31^{a}	13.60 ± 0.77^{a}
MDH	2.85 ± 0.13^{c}	3.27 ± 0.12^{b}	3.25 ± 0.29^{b}	3.72 ± 0.15^{a}
PPDK	3.91 ± 0.48^{b}	$4.54 \pm 0.26a^{a}$	4.29 ± 1.15^{ab}	4.32 ± 0.42^{ab}
PEPCK	4.22 ± 0.24^{d}	4.78 ± 0.07^{c}	5.60 ± 0.09^{a}	5.38 ± 0.27^{ab}

Abbrevations: PEPC, Phosphoenolpyruvate carboxylase; MDH, malate dehydroaenase; PPDK, pyruvate orthophosphate dikinase; PEPCK, phosphoenolpyruvate carboxykinase. n=4. Mean ± SD is presented. Different letters (a–d) indicate significant differences ($P < 0.05$) between lines.

Table S4. Photosynthetic parameters of CK and CBP lines.

Parameter	WT	CBP-1	CBP-2	CBP-3
Γ ($\mu mol\ mol^{-1}$)	56.68±0.81[a]	59.56±0.51[a]	58.86±0.72[a]	58.89±1.46[a]
A_{max} ($\mu mol\ CO_2\ m^{-2}\ s^{-1}$)	30.65±1.84[b]	34.67±0.49[a]	36.67±1.22[a]	35.42±0.60[a]
CE ($mol\ m^{-2}\ s^{-1}$)	0.119±0.0014[b]	0.130±0.0041[a]	0.152±0.0170[a]	0.132±0.0088[a]
R_{dark} ($\mu mol\ CO_2\ m^{-2}\ s^{-1}$)	-0.589±0.0059[b]	-0.903±0.054[a]	-0.915±0.140[a]	-0.857±0.1331[a]
AQY	0.0492±0.0011[a]	0.0638±0.0022[b]	0.0618±0.0008[b]	0.0593±0.0027[b]
LSP ($\mu mol\ m^{-2}\ s^{-1}$)	1716.07±18.37[a]	1698.55±25.08[a]	1661.23±7.71[a]	1655.75±40.77[a]
J_{max} ($\mu mol\ m^{-2}\ s^{-1}$)	162.12±4.64[b]	175.02±0.12[a]	169.76±1.36[ab]	172.56±2.03[a]
V_{cmax} ($\mu mol\ CO_2\ m^{-2}\ s^{-1}$)	71.67±1.70[b]	77.02±0.37[ab]	83.49±3.06[a]	82.62±2.54[a]

The CO_2 compensation point (CCP) and maximum carboxylation efficiency (CE) were calculated from the intercept and slope of the CO_2 response curves. Light response curves were acquired by increasing the photon flux density from 0 to 2000 $\mu mol\ m^{-2}s^{-1}$ at Ca 400 $\mu mol\ mol^{-1}$. Quantum yield for CO_2 assimilation (ΦCO_2) and respiration rates (Rd were calculated from the slope and intercept of the light-response curves (photon flux density < 100 $\mu mol\ m^{-2}s^{-1}$). Light-saturated photosynthetic rate (Amax) was calculated based on the light-response curves. Maximum carboxylation rate allowed by Rubisco (Vcmax) and rate of photosynthetic electron transport based on NADPH requirement (J) were determined by fitting the A-Ci response curves.

All measurements were performed using the flag leaf of CK and CBP lines at the filling stage, and the leaf temperature was set to 30°C.

Different letters (a–b) indicate significant differences (*P* < 0.05) between lines. n = 3. Mean ± SD is presented.

Table S5. Target metabolite contents of CK and CBP lines.

Metabolite content (Rel.amount mg FW^{-1})												
Name	BCBP-1	BCBP-2	BCBP-3	HCBP-1	HCBP-2	HCBP-3	BCK-1	BCK-2	BCK-3	HCK-1	HCK-2	HCK-3
Adenine	0.28753	0.30388	0.30541	0.24755	0.25079	0.25755	0.22609	0.17152	0.20804	0.20629	0.24573	0.22101
Alpha-Ketoglutaric acid	117.7546	259.7974	128.0726	205.8743	195.8855	130.5498	179.4861	165.64595	158.5478	182.6935	175.4834	173.83935
AMP	28.37501	31.01269	27.19698	15.99615	13.67428	8.57339	11.4841	7.7254	12.52005	13.39367	13.51761	11.72963
ATP	10.48172	8.84477	8.83409	5.43376	3.85117	3.02543	6.38653	6.14108	6.01403	3.39385	2.59171	2.3645
cis-Aconitic acid	75.19964	73.27095	71.06512	44.04234	38.54797	29.72857	49.32205	44.13887	44.26983	52.71494	66.91921	63.64303
Citric-acid	320.6686	532.104	368.73119	204.9347	194.5911	153.2645	294.4366	205.5548	250.0984	223.6091	186.6964	163.0162
D-Fructose-6-phosphoric acid	12.60421	20.89702	17.96104	12.17428	13.26932	9.34283	14.46648	12.66402	13.90485	10.61427	8.95017	9.08951
D-Glucose 6-phosphate	1.34995	2.66669	2.27298	0.28972	0.30749	0.17743	0.99699	1.09551	1.07788	0.40175	0.5316	0.40261
Dihydroxyacetone phosphate	7.9865	7.66207	7.09951	8.57443	13.93616	9.66956	9.1422	10.75238	7.44314	6.62114	5.68076	5.29651
Dl-Glyceric Acid	48.5282	63.53612	34.97574	49.12476	46.02653	37.02862	34.93391	24.43792	29.90008	62.53808	57.87667	59.19306
Flavin Mononucleotide	1.26918	1.42039	1.344785	1.29838	0.93012	0.74326	1.01667	1.032945	1.04922	0.9696	0.98714	0.97837
Fructose-1,6-bisphosphat e	19.5181	15.03845	10.93336	6.13777	4.49219	3.9963	8.56361	7.64518	6.85061	5.04582	4.64248	4.27417
Fumaric acid	39.94655	43.73069	42.74839	33.9647	29.14	24.40249	18.0269	16.00397	15.51745	54.59097	59.22274	66.88975
Glycerol 3-phosphate	12.28332	18.23275	15.08052	10.1768	10.54662	9.80698	13.56201	11.21922	12.05211	11.15111	12.57823	12.5585
Guanosine	6.98586	8.20051	6.15106	7.56945	5.53612	6.62504	7.48036	6.17282	7.42111	7.59251	6.43859	8.15242
IMP	0.26879	0.28432	0.25853	0.16102	0.18103	0.15547	0.25019	0.2475	0.21777	0.26675	0.27281	0.29822
Inosine	0.09373	0.1072	0.09435	0.05716	0.06225	0.05758	0.13441	0.08355	0.09016	0.08533	0.07689	0.07311
Isocitric acid	324.3528	374.0342	255.8072	181.3323	175.6154	169.6913	249.4137	206.6843	234.144	190.1077	175.3948	155.7169
Itaconic Acid	33.47453	32.82512	30.47612	20.76554	16.82381	13.12125	25.43711	20.7768	23.77198	33.03541	44.07128	39.73239

L-Alanine	6.64201	7.77629	6.34805	0.68031	0.85574	0.20517	2.52668	1.21498	1.10245	0.36043	0.38794	0.33551
L-Arginine	2.51165	3.34572	2.717455	7.02386	7.00004	5.10054	5.60919	5.72964	6.10527	2.65251	2.57721	2.74126
L-Asparagine	12.09454	17.3489	16.34422	11.79616	10.45641	11.17612	13.73038	12.79616	15.41319	9.82439	14.12497	9.79729
L-Citrulline	0.32015	0.23407	0.23535	0.19348	0.17943	0.17983	0.26573	0.21749	0.21231	0.04932	0.08329	0.06314
L-Cystine	5.85986	4.39756	3.26689	1.31802	1.02931	0.76219	1.9087	2.37913	1.52684	1.49416	1.38214	1.61565
L-Glutamic acid	35.12699	38.41542	31.06844	31.41612	27.71008	20.52737	22.21275	16.29925	22.36013	25.29895	23.27023	27.44515
L-Glutamine	24.03144	28.13358	25.03379	9.81484	9.13418	8.2852	13.7676	9.27225	12.71352	6.2852	6.33709	7.46874
L-Lactate	4.30719	5.52465	3.05243	4.42279	3.66373	4.86612	6.69598	7.03518	5.74644	3.75658	3.93813	2.94038
L-Ornithine	18.75435	14.44593	15.62445	18.89761	18.5114	18.83241	15.86872	13.57127	14.74411	9.93137	8.14533	10.0591
L-Serine	140.16334	143.96138	120.45027	84.16123	80.94356	79.60909	108.40344	103.91287	104.13717	69.4248	66.87275	65.46979
L-Threonine	7.48649	9.63572	7.06828	7.6572	7.93711	7.12722	8.32909	7.11561	8.21217	5.21976	4.61264	5.06976
L-Tyrosine	3.06059	3.56988	2.51187	1.82619	1.47733	1.40057	4.45176	3.30469	4.66854	1.96733	2.20027	2.77056
Malic Acid	790.4968	1213.67	879.3397	854.9503	732.1214	591.6586	541.0888	442.6779	441.8153	677.1994	699.2301	704.0909
Oxaloacetate	11.37303	20.0884	13.64033	6.16005	5.32987	6.2617	4.93468	4.59111	3.26452	3.59111	3.26452	2.8707
Phosphoenolpyruvic acid	2.40318	3.14737	2.56442	4.61404	3.69212	2.71835	2.15917	1.47117	2.41218	3.07328	1.44321	1.82804
Pyruvic acid	11.15091	14.32439	11.97165	10.6002	8.30191	10.63427	11.02108	9.48927	11.65745	8.13904	12.50239	10.71039
Succinic acid	12.11489	11.05619	12.03612	12.81925	11.6342	10.12125	1.12851	2.05017	1.96104	9.00767	10.02536	8.91423
UDP-GlcNAc	0.77886	1.05477	0.9598	0.51279	0.43954	0.35	0.45864	0.36958	0.45815	0.44425	0.44998	0.51676
UMP	1.98908	2.16374	2.01574	2.19846	1.61078	1.95562	1.65811	1.36539	1.63436	1.88569	2.27526	1.83717

Table S6. Comparison of agronomic trait between CK and CBP lines in 2023 in Lang fang. Each line at least 10 plants were counted.

Agronomic trait	CK	CBP-1	CBP-2	CBP-3
Plant height（cm）	112.25±1.29[a]	112.31±2.31[a]	114.06±1.9[a]	117.20±4.67[a]
Effectives tillers per plant	20±3[a]	20±2[a]	21±3[a]	22±1[a]
Panicle length (cm)	20.52±1.2[b]	21.48±1.02[a]	22.01±1.26[a]	21.24±0.85[a]
Biomass per plant（g）	112.3±0.6[b]	126.92±13.36[a]	129.8±7.72[a]	136.27±5.52[a]
Grain yield per plant (g)	38.4±1.7[b]	43.41±3.26[a]	42.60±3.47[a]	44.17±5.59[a]
1000-grain weight (g)	22.81±0.62[a]	23.40±0.91[a]	23.39±0.87[a]	24.42±0.81[a]
Grain number per panicle	119±6.93[b]	128±4.58[a]	133±6.21[a]	132±5.67[a]
Grain length (mm)	7.16±0.08[a]	7.20±0.30[a]	7.21±0.09[a]	7.22±0.15[a]
Grain width (mm)	3.01±0.07[a]	3.11±0.03[a]	3.12±0.05[a]	3.16±0.01[a]

Note: Different letters (a–b) indicate significant differences ($P < 0.05$) between lines.

Table S7. Primer sequences used in this study.

**Primes Name**	**SEQUENCE**
**Primers for overexpression intermediate vector construct**	
AcoPEPCK1-F	**ACAAGTTTGTACAAAAAAGCAGGC**TGCGGCCGCACTAGTCCCG
AcoPEPCK1-F	**CAACTTTATTATACAAAGTTG**CCTACCGGTGAGCTCTAGAATT
AcoPEPC1-F	**ACAAGTTTGTACAAAAAAGCAGGC**TTCTGCGGCCGCAAGCTTAAGCTGAGA
AcoPEPC1-R	**CAACTTTATTATACAAAGTTG**TAGCTCGAGATAGTTGTTGGGAGTAAT
AcoMDH1-F	ACAAAAAAGCAGGCT**GCGGCCGC**TAAGCTTGTACATGTCGACTAGTGTTTG
AcoMDH1-R	GGGCCCGGGACTAGT**GCGGCCGC**GGTAAATAGTCATTACTTTCTGAATGGA G
AcoCA1-F	AAAAAGCAGGCTTCT**GCGGCCGC**ATTAGCCTATCAATGTTCAATTTAATCC
AcoCA1-R	TCTCAGCTTAAGCTT**GCGGCCG**CTCTAGATGGAAAGAACAGGCTATGTC
AcoPPDK1-F	**CAACTTTGTATAATAAAGTTG**TTACTAGTGCGGCCGCAAGCTT
AcoPPDK1-R	**AGCCTGCTTTTTTGTACAAACT**TGTGCGGCCGCGGTTGTTGGTT
AtALMT9-F	TTAATTGGATCGTTGATAACTTCGTATAATGTATGCTATACGAAGT
AtALMT9-R	GCTACCTTAGGACCGAAGCTTTCCCTGACACAAAAAGCCTATACTG
**Target site primers for CRISPR vector construct**	
OsU6c-F	GTAAGGCGCGCCAATGATACCGCTCATTAGCGGTATGCATGT
OsSLAC-R	AGGGAGACCTGCCGGCTGAAGCCTCAGCGCAGCAGCTTAT
OsSLAC-F	TTCAGCCGGCAGGTCTCCCTGTTTTAGAGCTAGAAATAGCAA
gRNA-R	AGAGGCGCGCCGTAGTGCTCGTCCATCCACTCCAAGCTCT
**Primers for subcellular localization**	
AcoPEPC1-GFP-F	GGACAGCCCAGATCACTAGTATGGCGAAGAGTTCGGTGGA
AcoPEPC1-GFP-R	AGATCTCTGCAGAGCTCCTGGTCCTAACTCCTTGTGACCA
AcoMDH1-GFP-F	GGACAGCCCAGATCACTAGTATGGCGAAGGATCCCGTACG
AcoMDH1-GFP-R	AGATCTCTGCAGAGCTCCTGATCGCATGAGAACGGAGAGA
AcoPPDK1-GFP-F	GGACAGCCCAGATCACTAGTGAAACGTGCATCTAATAGTA
AcoPPDK1-GFP-R	AGATCTCTGCAGAGCTCCTGGGCGACCACCTGGGCCGCTG
AcoCA1-GFP-F	GGACAGCCCAGATCACTAGTATGTCGACCGCCGCGGCGA
AcoCA1-GFP-R	AGATCTCTGCAGAGCTCCTGGAAGCTCTGAACCCTCTAGTT
AcoPEPCK1-GFP-F	GGACAGCCCAGATCACTAGTATGGAGGCGGAGACCGGGGA
AcoPEPCK1-GFP-R	AGATCTCTGCAGAGCTCCTGAGCGACGACCGGGTTTGAAC
AtALMT9-GFP-F	AGGACAGCCCAGATCACTAGTATGGCGGCGAAGCAAGGT
AtALMT9-GFP-F	GGTCCTCGAGACGTCTCTAGACATCCCAAAACACCTACGAATCT
**Primers for gene-specific identification of transgenic lines**	
jdAcoCA1-F	TGAGATCGGGGTTCGACTACT
jdAcoCA1-R	GGACACATTCACAGCCTCCT
jdAcoPEPC1-F	GGCAACTTATGAGAGCGCAA
jdAcoPEPC1-R	CGCATTTCATCCTGTGGAGC
jdAcoPPDK1-F	TGGTCAAGATGGCCTGGTTC
jdAcoPPDK1-R	GAAGCAGCTGTATTTGGCGG
jdAcoPEPCK1-F	GAGCACACTCGTGAAGTGGA

jdAcoPEPCK1-R	GCACTCCTTCCAGCTCAGTT
jdAcoMDH1-F	TCGAGGCTTGTAATGGGGTC
jdAcoMDH1-R	GTACCCTCAGGTGTCCCAAG
jdALMT9-F	ACGATCCATAGCAAGCCCAG
jdALMT9-R	CTACGCCCACTCTCTGAAGT
C-SLAC1F	TCTTGGGAGCTCACGTACGT
C-SLAC1R	AGAAGAAGTTGACGCGCACC
**Primers used for qRT-PCR**	
qAcoMDH1-F	GCTGTGATGGTCGGTGGATT
qAcoMDH1-R	CGGGATGGATGGAGCAAACT
qAcoPEPCK1-F	GTCTGTGGACAGTGTGGTCT
qAcoPEPCK1-R	ATGGTTATCCTCGGCACACA
qAcoPPDK1-F	GCATCCAGAAACGCAACACT
qAcoPPDK1-R	GGTCAAGTTGTCTTCACGGC
qAcoCA1-F	GGCAAAGCCCGAAGTTTATGG
qAcoCA1-R	ACCTTGAGTTGGATGACCGC
qAcoPEPC1-F	GCAACTTATGAGAGCGCAAGC
qAcoPEPC1-R	ATAGCTAAGGCCTCATCGCC
qACTIN-F	TGCTATGTACGTCGCCATCCAG
qACTIN-R	AATGAGTAACCACGCTCCGTCA
qALMT9F	GGAAAGCTTGGGAGATGGGAG
qALMT9R	GCCATTCCAAGAGCAAGACCTC
UBQ-F	AACCAGCTGAGGCCCAAGA
UBQ-R	ACGATTGATTTAACCAGTCCATGA
